# Metabolic status of CSF distinguishes rats with tauopathy from controls

**DOI:** 10.1186/s13195-017-0303-5

**Published:** 2017-09-21

**Authors:** Radana Karlíková, Kateřina Mičová, Lukáš Najdekr, Alžběta Gardlo, Tomáš Adam, Petra Majerová, David Friedecký, Andrej Kováč

**Affiliations:** 1 Institute of Molecular and Translational Medicine, Faculty of Medicine and Dentistry, Palacký University Olomouc, Hněvotínská 5, 779 00 Olomouc, Czech Republic; 2 Department of Clinical Biochemistry, University Hospital Olomouc, I. P. Pavlova 6, 775 20 Olomouc, Czech Republic; 3 Laboratory for Inherited Metabolic Disorders, Faculty of Medicine and Dentistry, Palacký University Olomouc, I. P. Pavlova 6, 775 20 Olomouc, Czech Republic; 4 Institute of Neuroimmunology, Slovak Academy of Sciences, Dúbravská cesta 9, 84510 Bratislava, Slovak Republic; 5 AXON Neuroscience R&D, Dvořákovo nábrežie 10, 811 02 Bratislava, Slovak Republic

**Keywords:** Tauopathy, Transgenic rat model, Tau protein, Metabolomics

## Abstract

**Background:**

Tauopathies represent heterogeneous groups of neurodegenerative diseases that are characterised by abnormal deposition of the microtubule-associated protein tau. Alzheimer’s disease is the most prevalent tauopathy, affecting more than 35 million people worldwide. In this study we investigated changes in metabolic pathways associated with tau-induced neurodegeneration.

**Methods:**

Cerebrospinal fluid (CSF), plasma and brain tissue were collected from a transgenic rat model for tauopathies and from age-matched control animals. The samples were analysed by targeted and untargeted metabolomic methods using high-performance liquid chromatography coupled to mass spectrometry. Unsupervised and supervised statistical analysis revealed biochemical changes associated with the tauopathy process.

**Results:**

Energy deprivation and potentially neural apoptosis were reflected in increased purine nucleotide catabolism and decreased levels of citric acid cycle intermediates and glucose. However, in CSF, increased levels of citrate and aconitate that can be attributed to glial activation were observed. Other significant changes were found in arginine and phosphatidylcholine metabolism.

**Conclusions:**

Despite an enormous effort invested in development of biomarkers for tauopathies during the last 20 years, there is no clinically used biomarker or assay on the market. One of the most promising strategies is to create a panel of markers (e.g., small molecules, proteins) that will be continuously monitored and correlated with patients’ clinical outcome. In this study, we identified several metabolic changes that are affected during the tauopathy process and may be considered as potential markers of tauopathies in humans.

**Electronic supplementary material:**

The online version of this article (doi:10.1186/s13195-017-0303-5) contains supplementary material, which is available to authorized users.

## Background

Tauopathies include around 20 different degenerative disorders, such as Alzheimer’s disease (AD), progressive supranuclear palsy, Pick’s disease, corticobasal degeneration, frontotemporal dementia with Parkinsonism linked to chromosome 17, dementia pugilistica/traumatic brain injury/chronic traumatic encephalopathy complex, and others. According to the pathology, we divide tauopathies into three main groups: tauopathies associated with the deposition-predominant tau pathology composed mainly of abnormal deposition of tau into intracellular neurofibrillary tangles, tauopathies associated with the deposition of amyloid beta (Aβ), and tauopathies associated with other pathologies [1].

Currently, 46.8 million people are living with various forms of dementia worldwide, and this number is rapidly increasing every year [2]. Moreover, there is no effective therapeutic strategy, which highlights the importance of further intensive research, including ‘omics’ studies with transgenic (Tg) animal models. Among tauopathies, AD is the most common chronic, irreversible neurodegenerative disease that affects higher structures of the brain, causing the clinical symptoms known as dementia. AD is characterised by cerebrovascular and neuronal dysfunction leading to a progressive decrease in cognitive function. On the histopathological level, AD is defined by the presence of extracellular amyloid plaques composed of Aβ peptide aggregates and neurofibrillary tangles formed of tau protein.

Tau is a highly soluble microtubule-associated protein localised predominantly in neuronal axons. In AD and other tauopathies, tau undergoes changes that include posttranslational modifications such as hyperphosphorylation and truncation, which lead to aggregation and deposition of tau into insoluble structures called neurofibrillary tangles [3].

In our present study we used the SHR72 Tg rat model for tauopathy expressing truncated tau protein 151-391/4R. Previously, we found that removing the C- and N-terminal parts of tau protein triggered the misfolding cascade and the development of progressive neurofibrillary pathology in Tg animals. The SHR72 Tg rat model recapitulates many features seen in AD and other tauopathies, including tau hyperphosphorylation and truncation, formation of neurofibrillary tangles located in the brainstem, insoluble tau complexes [4], white matter damage [5], increased reactive oxygen species, and mitochondrial damage [6, 7]. All these pathological changes are accompanied by extensive neuroinflammation [8]. These findings make the SHR72 Tg rat model an important tool for AD research; it has already proven its value in the study of the first anti-tau vaccine therapy [9]. Identification of molecular markers associated with neuronal changes in our Tg rat model would aid the understanding of individual cellular processes affected by tau-induced neurodegeneration.

Several metabolomic studies were conducted on Tg mouse models of AD expressing mutated amyloid precursor protein (APP), mutated presenilin-1 or -2, or a combination of both. The researchers in these studies reported changes in production of neurotransmitters such as glutamate, glutamine, *N*-acetylaspartate, aspartate or gamma-aminobutyric acid in the brain areas affected by pathology. Some studies indicated changes in energy metabolism, demonstrated by a decrease in adenosine 5′-triphosphate (ATP), adenosine 5′-diphosphate (ADP) and nicotinamide adenine dinucleotide [10]. It is not known, however, how these changes or which of them are reflected in body fluids such as plasma and cerebrospinal fluid (CSF), which are used for clinical diagnosis in humans. Fukuhara et al. [11] used a nuclear magnetic resonance-based approach to study urinary metabolites on the mutant tau/APP Tg mouse model. They detected changes in oxidative stress metabolites, confirming the role oxidative stress in AD pathogenesis.

In human metabolomic studies related to neurological diseases, CSF and plasma are the usual body fluids analysed. Analysis of CSF from 79 patients with AD (early and late stages of disease) and 51 control subjects revealed significant changes of 5 unknown and 11 known analytes. Cysteine and uridine were evaluated as the most significant metabolites for AD prediction [12]. A recent report highlighted the value of plasma metabolomic profiling in neurodegeneration, especially in AD. Liquid chromatography-mass spectrometry (LC-MS)-based analysis revealed several potential biomarkers between the healthy subjects and individuals with mild cognitive impairment (MCI)/AD. Previous quantitative experiments using commercially available assays confirmed lipids as the best phenoconversion predictors [13, 14]. A large cohort study using the same approach was less conclusive, however [15]. Other plasma metabolites, such as amino acids and bile acids, also showed very promising results [16].

We present the results of our metabolomic study with the SHR72 Tg rat model for tauopathies using high-performance liquid chromatography coupled to triple-quadrupole or Orbitrap mass spectrometry (Thermo Fisher Scientific, Waltham, MA, USA). In comparison with previously conducted studies with Tg animal models of AD and other tauopathies, we took advantage of rat and, except for the brain tissue, we simultaneously analysed CSF and plasma from the same animals. We demonstrate, for the first time to our knowledge, that tau pathology alone can initiate changes in endogenous metabolic pathways that can be detected in clinically useful biological materials.

## Methods

### Animals

The generation and characterisation of a Tg rat model for tauopathy expressing human truncated tau (amino acids 151–391/4R) are described in detail elsewhere [4]. For the pilot study, heterozygous Tg rats (SHR72, 5–6 months old, *n* = 10) and non-Tg SHR age-matched controls (SHR, 5–6 months old, *n* = 10) were used. For the confirmatory study, seven Tg and six age-matched control rats were used. All animals were housed under standard laboratory conditions with free access to water and food and were kept under diurnal lighting conditions (12-h/12-h light/dark cycle with light starting at 7:00 a.m.). All animal experiments were carried out according to the institutional animal care guidelines and in conformity to international standards (Animal Research: Reporting of In Vivo Experiments guidelines), and they were approved by the State Veterinary and Food Administration of the Slovak Republic (Ro-1101/14-221C) and by the Ethics Committee of the Institute of Neuroimmunology, Slovak Academy of Sciences. Efforts were made to minimise the number of animals used and to limit discomfort, pain or any other suffering of the experimental animals used in this study.

### Collection of cerebrospinal fluid, plasma and brain tissue

CSF for both studies (pilot and confirmation) was collected from the cisterna magna. Animals were anaesthetised with a tiletamine/zolazepam/xylazine mixture and fixed in a head holder, and a midline incision in the skin was made up to the head area to permit easy access to the cisterna magna. Approximately 80 μl of CSF was collected from each animal. After a short centrifugation step (3 minutes at 5000 × *g*, 4 °C), all CSF samples were immediately flash-frozen in liquid nitrogen and stored at −70 °C until used. The blood was collected from the heart. Approximately 4 ml of the blood was collected using a 22-gauge needle, then it was centrifuged for 10 minutes at 5000 × *g* (4 °C). Plasma was collected, flash-frozen in liquid nitrogen and stored at −80 °C until used. The brain tissue from the brainstem was flash-frozen in liquid nitrogen and stored at −80 °C until used.

### Sample preparation

CSF and plasma samples were prepared by protein precipitation. Aliquots (50 μl) of each sample were treated with 150 μl of methanol, vortexed and kept overnight at −80 °C. After centrifugation (6700 × *g*, 15 minutes, 4 °C), 150 μl of supernatant was transferred into a glass vial and used for analysis, and 10 μl were used for quality control (QC) sample preparation based on pooling of equal aliquots of plasma or CSF sample extracts. The brain tissue was homogenised on dry ice using the FastPrep-24 instrument (MP Biomedicals, Santa Ana, CA, USA) in 80% (vol/vol) gradient grade methanol (cooled down to −80 °C) (Sigma-Aldrich, St. Louis, MO, USA). The mixture was centrifuged at 14,000 × *g* for 10 minutes (4 °C) and SpeedVac (Thermo Fisher Scientific) freeze-dried to a pellet using no heat. The pellet was re-suspended in 100 μl of 50% methanol (vol/vol), sonicated for 10 minutes, vortexed and centrifuged (6700 × *g*, 15 minutes, 4 °C). Eighty microliters of supernatant was transferred into the glass vial, and 10 μl was used for QC sample preparation. All samples were used immediately for analysis and then stored at −50 °C.

### Extraction of sarkosyl insoluble tau

Sarkosyl-insoluble tau was isolated from the brainstem of 5- to 6-month-old rats. Frozen brain samples of the studied rats were homogenised in 10 vol of ice-cold extraction buffer (SL buffer: 20 mM Tris, pH 7.4; 800 mM NaCl; 1 mM ethylene glycol tetraacetic acid), 1 mM ethylenediaminetetraacetic acid (EDTA), 0.5% β-mercaptoethanol, 10% sucrose, 1 mM Na_3_VO_4_, 20 mM NaF, supplemented with EDTA-free protease inhibitor cocktail tablet (Roche Diagnostics, Indianapolis, IN, USA) using an OMNI TH tissue homogeniser (OMNI International, Kennesaw, GA, USA). After 5-minute incubation on ice, the homogenates were cleared by centrifugation at 20,000 × *g* for 20 minutes at 4 °C. Solid sarkosyl (*N*-lauroyl sarcosine, Na-salt; Sigma-Aldrich) was added to the supernatant to achieve 1% concentration and stirred for 1 h. Thereafter it was centrifuged at 100,000 × *g* for 1.5 h at room temperature. The supernatant was collected, and pellets were gently rinsed with 1 ml of the SL buffer and centrifuged for 20 minutes at room temperature. The pellets were dissolved in sodium dodecyl sulphate (SDS) sample loading buffer.

### Western blot analysis

Sarkosyl-insoluble tau proteins purified from brain were run on 12% SDS-polyacrylamide gels and transferred onto nitrocellulose membranes in 10 mM *N*-cyclohexyl-3-aminopropanesulphonic acid buffer (pH 11; Carl Roth, Karlsruhe, Germany). The membranes were blocked in 5% milk in Tris-buffered saline/Tween 20 (137 mM NaCl, 20 mM Tris-HCl, pH 7.4, 0.1% Tween 20) for 1 h and incubated overnight with primary DC25 antibody (AXON Neuroscience SE, Bratislava, Slovakia), which recognises residues 347–354 of the longest human tau isoform, Tau40. Incubation with primary antibodies was followed by incubation with horseradish peroxidase-conjugated secondary antibody (Dako, Mississauga, ON, Canada). Enhanced chemiluminescence Western blots were digitised with the LAS-3000 charge-coupled device imaging system (FUJIFILM, Tokyo, Japan). Densitometric data analysis and relative quantification of Western blots were performed using the AIDA BioPackage programme (Raytest, Straubenhardt, Germany).

### Metabolomic analysis

#### Targeted metabolomic analysis

Targeted metabolic analysis was performed by liquid chromatography-tandem mass spectrometry using the UltiMate 3000 Rapid Separation system (Dionex, Sunnyvale, CA, USA) coupled to a triple-quadrupole mass spectrometer (Triple Quad 6500; SCIEX, Framingham, MA, USA). Detailed information on the chromatographic and mass spectrometry methods we used is provided in previously published work [17]. The separation was carried out on an aminopropyl column (Luna 3-μm NH2, 2 × 100 mm; Phenomenex, Torrance, CA, USA) maintained at 35 °C. Mass spectrometer settings were adjusted to a new-generation mass spectrometer (Triple Quad 6500). The parameters of the ion source and gases were set at ion spray voltage +5500 V and −4500 V, curtain gas 40 psi, both ion source gases 40 psi, and source temperature 400 °C. Evaluation of data was performed using MultiQuant 3.0 software (SCIEX) and statistically processed. Abbreviations of metabolites from targeted metabolomic analysis are shown in Additional file 1: Table S1.

#### Untargeted metabolomic analysis

Data for untargeted metabolomic analysis were measured using a liquid chromatography–high-resolution mass spectrometry system consisting of the UltiMate 3000 Rapid Separation system and an LTQ Orbitrap Elite mass spectrometer (Thermo Fisher Scientific) controlled using Chromeleon Xpress 6.80 software, Dionex DCMSLink 2.12 software and Xcalibur 2.2 SP1 software (Thermo Fisher Scientific). The chromatographic method was adjusted according to the work of Nygren et al. [18]. The separation was performed with an ethylene bridged hybrid (BEH) C18 1.7-μm column (2.1 × 100 mm; Waters, Milford, MA, USA) protected by a BEH Shield RP18 1.7-μm guard column (5 × 2.1 mm; Waters). The mobile phase consisted of 1% 1 M ammonium acetate with 0.1% formic acid in water (A) and in acetonitrile/isopropyl alcohol (1:1 vol/vol) (B). The gradient was programed as follows: 0–2 minutes, 35 → 80% B; 2–7 minutes, 80 → 100%; 7–14 minutes, 100% B. Then, in 0.5 minute, the system was set to initial conditions and equilibrated for 3.5 minutes. The whole analysis took 18 minutes. The column was maintained at 50 °C, and the flow rate was set at 0.3 ml/minute. The Orbitrap Elite mass spectrometer was operated in positive full-scan mode within a mass-to-charge ratio (*m/z*) range of 200–1200 and at 120,000 FWHM resolution. Electrospray ion source parameters were set at follows: heater temperature 250 °C, sheath gas 35 AU, auxiliary gas 15 AU, source voltage +3 kV and capillary temperature 300 °C. A Thermo Tune Plus 2.7.0.1103 SP1 (Thermo Fisher Scientific) was used as instrument control software, and data were acquired using Thermo Xcalibur 2.2 SP1.48 software (Thermo Fisher Scientific). Errors for all measurements were below 2 ppm.

Identification of the most discriminant features was performed by fragmentation of specific *m/z* in positive and negative modes with collision-induced dissociation and higher-energy collisional dissociation fragmentation using the Orbitrap Elite mass spectrometer. Because of low sensitivity of the instrument in negative mode, the fragmentation study was transferred to the triple-quadrupole Triple Quad 6500 system. Data were acquired in product ion mode of the most discriminant parent molecule with negative electrospray ionisation in the range *m/z* 70 up to *m/z* 1050, and simultaneously positive multiple reaction monitoring (MRM) mode for the peak retention time confirmation for each feature was used. The MRM transition was set to *m/z* of the parent molecule and the most intensive fragment obtained by the molecule fragmentation with the Orbitrap instrument in positive mode. Ion source parameters were set at ion spray voltage ±4500 V, curtain gas 40 psi in positive and 30 psi in negative mode, both ion source gases 40 psi, and source temperature 400 °C. The mass spectrometer settings were declustering potential ±100 V, entrance potential ±10 V, collision energy +30 or −35 V, and collision exit potential +13 or −16 V. For lipids, the structure identification-obtained fragmentation spectra were compared with LIPID MAPS and Merlin database data. The level of metabolite identification was determined using the Metabolomics Standards Initiative (MSI) system, ranging from level 2 to level 4. For MSI level 2 (‘putatively annotated compounds’), identification was done by comparison of exact mass and fragmentation spectra with databases. For MSI level 3 (‘putatively characterised compound classes’), only the main class of compound was determined by exact mass and fragmentation spectra. For MSI level 4, (‘unknown compounds’), the acquired spectra from fragmentation were not found in databases [19].

### Statistical analysis

The R programme (version 3.1.2) was used for data treatment and statistical evaluation [19, 20]. In untargeted analysis, the peak finding was obtained using the XCMS package with the centWave method [21]. The CAMERA package was applied to data structure for identification of isotopic patterns and adducts [19, 22]. Zero values were replaced by integration of noise in the same retention time using the ‘fillPeaks’ XCMS function. Further data treatment was the same for both analyses. The quality control-based, locally estimated smoothing signal correction was applied on the datasets for all biological materials [19, 23, 24]. On the basis of coefficients of variation (CVs) calculated from the QC samples, metabolites/features with a CV higher than 30% were excluded from further data processing. The data were analysed as compositional using centred log ratio (clr) transformation and mean centring [25]. The *p* value was calculated by *t* test, and then the Bonferroni correction was applied (α = 0.05/number of metabolites; e.g., for CSF samples, α = 0.05/96). Corrected α values for every biological material are shown in the labels of Table 1 for targeted analysis and in Table 2 for untargeted metabolomic analysis. For confirmation, study α values are shown in Additional file 1: Tables S2 and S3. Box plots of all metabolites/features were constructed for every biological material.

**Table 1:** Twenty most discriminating metabolites from orthogonal projections to latent structures discriminant analysis (sorted by absolute value of pcorr1 axis; variation related to variable magnitude)

Cerebrospinal fluid					Plasma					Brain tissue				
Name	p1	pcorr1	Fold change	*p* Value (α = 5.21E-04)^a^	Name	p1	pcorr1	Fold change	*p* Value (α = 3.07E-04)^a^	Name	p1	pcorr1	Fold change	*p* Value (α = 2.06E-04)^a^
Tr	−0.91	−0.95	0.64	1.68E-07	Glutamine	0.97	0.93	1.19	1.37E-03	NaMN	2.15	0.95	1.52	4.44E-03
Citrate_isocitrate	−1.06	−0.93	0.52	8.71E-08	Proline	1.39	0.91	1.43	1.74E-03	CDP	4.47	0.95	6.36	2.94E-02
Carnitine	−0.78	−0.9	0.76	2.90E-05	Spermine	1.38	0.9	1.45	6.81E-04	ATP	4.12	0.93	6.55	4.47E-02
Myo-inositol	0.77	0.86	1.38	1.29E-04	Creatinine	1.5	0.89	1.67	3.33E-06	ADP	3.24	0.92	2.53	7.97E-02
Deoxyuridine	−0.6	−0.82	0.83	2.66E-04	DHA	−1.59	−0.89	0.7	4.67E-03	dGDP	3.22	0.92	2.27	6.39E-02
Cytosine	−0.61	−0.8	0.79	6.89E-04	Citrulline	1.43	0.87	1.49	1.08E-02	3PG	3.36	0.91	2.75	9.99E-02
S-ArMet	0.57	0.75	1.23	3.59E-03	C12	−1.75	−0.84	0.6	8.70E-02	Xanthine	−3.34	−0.9	0.34	2.63E-02
Creatinine	0.5	0.75	1.12	1.40E-03	C14:1OH	−1.58	−0.82	0.71	6.03E-02	UDP	4.03	0.9	5.1	5.52E-02
Ribose	0.45	0.71	1.15	9.23E-04	mHis_NmHis	−1.33	−0.82	0.73	2.60E-03	Hx	−3.38	−0.9	0.34	2.22E-01
mHis_NmHis	−0.63	−0.7	0.68	8.27E-03	NAcGlcnh2_NAcGalnh2_NAcManh2	1.23	0.82	1.42	2.80E-03	IDP	3.08	0.89	2.18	1.15E-01
Allantoin	−0.6	−0.69	0.82	2.91E-03	Succ_mma	1.44	0.81	1.32	1.24E-02	Inosine	−3.49	−0.89	0.28	1.98E-01
hCar	0.47	0.69	1.22	2.25E-02	Arabitol_ribitol	1.69	0.8	1.55	8.61E-02	dimGly_nh2isobut	−2.52	−0.88	0.51	1.84E-01
Glucose	0.46	0.68	1.2	2.19E-03	Trigonelline	1.85	0.79	1.58	4.00E-02	Uracil	−2.9	−0.88	0.47	2.44E-01
Succ_mma	0.36	0.66	1.08	4.89E-03	C14	−1.6	−0.78	0.76	1.48E-01	rib5P	−3.16	−0.86	0.34	2.62E-01
Aconitate	−0.54	−0.64	0.78	1.42E-02	oPro_pipec	0.82	0.78	1.15	7.67E-03	PCr	4.38	0.86	4.81	2.69E-01
Uridine	0.45	0.63	1.19	4.74E-03	C14	−1.53	−0.76	0.68	1.18E-01	Niacin	−1.93	−0.85	0.63	1.63E-01
Valine	−0.4	−0.59	0.83	4.95E-02	C14:1	−1.52	−0.76	0.58	8.49E-02	GuaBut	2.35	0.85	1.88	2.59E-01
Phenyl acetate	0.8	0.59	2.05	3.72E-02	C18:1	−1.47	−0.75	0.66	1.36E-01	acMet	−2.78	−0.85	0.54	3.10E-01
Fructose	0.4	0.59	1.2	1.69E-02	C16	−1.62	−0.75	0.72	1.26E-01	NADP	3.67	0.84	2.27	1.94E-02
C5	−0.5	−0.57	0.69	2.71E-02	C16OH	−1.4	−0.74	0.73	1.49E-01	Tr	−2.3	−0.84	0.6	2.04E-02

*Abbreviations: 3PG* Glycerate 3-phosphate, *acMet N*-acetylmethionine, *C12* Dodecanoylcarnitine, *C14* Tetradecanoylcarnitine, *C14:1* Tetradecenoylcarnitine, *C14:1OH* Hydroxytetradecenoylcarnitine, *C16* Hexadecanoylcarnitine, *C16:1OH* Hydroxyhexadecenoylcarnitine, *C16OH* Hydroxyhexadecanoylcarnitine, *C18:1* Octadecenoylcarnitine, *C5* Valerylcarnitine, *CDP* Cytidine 5′-diphosphate, *dGDP* Deoxyguanosine 5′-diphosphate, *DHA* Docosahexaenoic acid, *dimGly_nh2isobut* Dimethylglycine/2-aminoisobutyric acid, *GuaBut* Guanidinobutanoate, *hCar* Homocarnosine, *Hx* Hypoxanthine, *IDP* Inosine 5′-diphosphate, *mHis_NmHis* 3-Methylhistidine/*N*-methylhistidine, *NAcGlcnh2_NAcGalnh2_NAcManh2 N*-acetylglucosamine/*N*-acetylgalactosamine/*N*-acetylmannosamine, *NADP* Nicotinamide adenine dinucleotide phosphate, *NaMN* Nicotinamide mononucleotide, *oPro_pipec* 5-Oxoproline/ pipecolate, *PCr* Phosphocreatine, *rib5P_xyl5P* Ribose 5-phosphate/xylulose 5-phosphate, *S-ArMet S*-adenosylmethionine, *Succ_mma* Succinate/methylmalonate, *Tr* Thymidine, *UDP* Uridine 5′-diphosphate

*p* Values and fold changes for metabolites are shown

^a^Corrected α value (after Bonferroni correction)

**Table 2:** Discriminating features from orthogonal projections to latent structures discriminant analysis with identification of lipid structure

Name	p1	pcorr1	Fold change	*p* Value (α = 1.28E-04)^a^	Ion	*m/z*	Error ppm	Formula	MSI level
Cerebrospinal fluid									
PC(P-36:3)/PC(O-36:4)	−4.44	−0.98	0.22	1.96E-10	[M + H]^+^	768.5901	−0.13	C44H83NO7P	3
PC	−3.54	−0.98	0.36	8.43E-10	[M + H]^+^	703.5747	–	–	3
PC(17:0/18:1)/PC(35:1)	−3.96	−0.98	0.28	1.66E-09	[M + H]^+^	774.6004	−0.43	C43H85O8NP	2
PC(P-36:4)/PC(O-36:5)	−3.65	−0.97	0.29	5.08E-08	[M + H]^+^	766.574	−0.65	C44H81NO7P	3
PC(16:0/18:2)/PC(34:2)	−3.41	−0.97	0.39	6.21E-08	[M + H]^+^	758.5691	−0.4	C42H81O8NP	2
LysoPC(18:1)	−3.42	−0.96	0.39	1.13E-07	[M + H]^+^	522.3553	−0.19	C26H53O7NP	2
PC(18:0/18:1)/PC(36:1)	−2.98	−0.96	0.51	1.04E-06	[M + H]^+^	788.6161	−0.36	C44H87O8NP	2
PC(16:0/18:1)/PC(34:1)	−2.86	−0.96	0.53	1.31E-06	[M + H]^+^	760.5847	−0.5	C42H83O8NP	2
PC(P-36:2)/PC(O-36:3)	−4.35	−0.95	0.19	4.26E-07	[M + H]^+^	770.6056	−0.25	C44H85NO7P	3
PC(16:0/20:4)/PC(36:4)	−3.03	−0.95	0.46	9.59E-07	[M + H]^+^	782.567	−3.07	C44H81O8NP	2
Unknown compound	−2.78	−0.95	0.57	1.42E-05	[M + H]^+^	850.5542	–	–	4
PC(C18:0/20:4)/PC(38:4)	−2.54	−0.95	0.57	1.44E-05	[M + H]^+^	811.6043	–5.25	C46H86O8NP	2
PC	−3.9	−0.94	0.31	2.95E-08	[M + H]^+^	813.6836	–	–	3
PC(16:0/16:1)/PC(32:1)	−2.7	−0.93	0.39	1.67E-07	[M + H]^+^	732.5537	−0.11	C40H79O8NP	2
PC(16:0/16:0)/PC(32:0)	−2.7	−0.93	0.51	1.89E-05	[M + H]^+^	734.5692	−0.32	C40H81O8NP	2
PC(16:0/18:3)/PC(16:1/18:2)/PC(34:3)	−2.78	−0.92	0.5	2.88E-05	[M + H]^+^	756.5513	−3.28	C42H79O8NP	2
PC	−2.2	−0.91	0.66	9.11E-05	[M + H]^+^	731.6058	–	–	3
LysoPC(16:0)	−3.01	−0.89	0.44	9.53E-05	[M + H]^+^	496.3397	−0.2	C24H51O7NP	2
Name	p1	pcorr1	fold change	*p* Value (α =6.56E-05)^a^	ion	*m/z*	error ppm	formula	MSI level
Plasma									
PC(42:9)	−3.51	−0.93	0.63	2.21E-04	[M + H]^+^	856.5815	−4.2	C50H83NO8P	3
PC(18:0/22:6)/PC(18:1/22:5)/PC(40:6)	−3.37	−0.93	0.66	1.35E-04	[M + H]^+^	834.5997	−1.19	C48H85NO8P	2
PC	−3.01	−0.92	0.68	1.16E-04	[M + H]^+^	807.571	–	–	3
PC	−4.1	−0.92	0.54	1.08E-04	[M + H]^+^	829.0515	–	–	3
PC(20:2/0:0)	4.01	0.9	1.63	4.33E-03	[M + H]^+^	548.3705	−1.09	C28H55O7NP	2
PC	−3.08	−0.9	0.79	8.78E-03	[M + H]^+^	792.6278	–	–	3
PC(18:0/18:1)/PC(36:1)	−2.6	−0.88	0.82	1.14E-02	[M + H]^+^	788.6164	−0.03	C44H87NO8P	2
PC	−2.91	−0.87	0.74	1.56E-03	[M + H]^+^	892.6534	–		3
PC	−3.53	−0.86	0.64	6.31E-04	[M + H]^+^	859.59	–	–	3
PC	−3.34	−0.83	0.7	1.30E-03	[M + H]^+^	992.5569	–	–	3
PC(P-40:3)/PC(O-40:4)	−2.61	−0.82	0.73	5.59E-03	[M + H]^+^	824.6524	−0.48	C48H91NO7P	3
PC	−2.68	−0.81	0.87	5.91E-02	[M + H]^+^	764.5976	9.88	C41H83O9NP	3
PC(16:0/16:0)/PC(32:0)	−2.12	−0.79	0.9	5.38E-02	[M + H]^+^	734.5702	1.09	C40H81O8NP	2
LysoPC(20:3)	2.61	0.77	1.2	1.20E-01	[M + H]^+^	546.3546	−1.46	C28H53O7NP	2
PC(20:0/0:0)	2.75	0.77	1.3	6.72E-03	[M + H]^+^	552.402	−0.72	C28H59O7NP	2
LysoPC(22:5)	3.15	0.76	1.39	7.30E-02	[M + H]^+^	570.3545	−1.58	C30H53NO7P	2

*Abbreviations: MSI* Metabolomics Standards Initiative, *m/z* Mass-to-charge ratio, *PC* Phosphatidylcholine

*p* Values and fold change for these features are depicted

^a^ Corrected α value (after Bonferroni correction)

The fold change value was calculated as exp[med(SHR)-med(SHR72)], where med denotes median and exp is exponential, because clr transformation was used. Unsupervised principal component analysis (PCA) and supervised orthogonal projections to latent structures discriminant analysis (OPLS-DA) were applied on data. PCA works without information about to which group the samples belong, in contrast to OPLS-DA, where groups are defined. An OPLS-DA S-plot was used for visualisation of the metabolites most responsible for the group separation. The covariance (the contribution of the magnitude of the model component scores) is represented by the *x*-axis (p1), and correlation (the reliability of the model component scores) is represented by the *y*-axis (pcorr1).

## Results

### Analysis of paired helical filament tau

Tau pathology in the brainstem of Tg animals was analysed using biochemical Western blot analysis. There were no significant differences in sarkosyl-insoluble tau between individual Tg animals (Additional file 1: Figure S1).

### Targeted metabolomic analysis

**Fig. 1:**
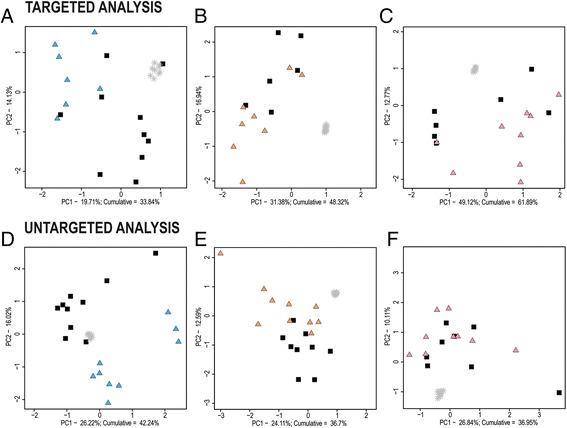
Principal component analysis score plots of targeted and untargeted metabolomic analyses. Cerebrospinal fluid (**a**, **d**), plasma (**b**, **e**) and brain tissue samples (**c**, **f**) of controls (*black squares*) and transgenic rats (*blue, orange* and *pink triangles*). *Grey stars* represent quality control samples. *PC* Principal component

Using targeted metabolomic analysis, 96 metabolites were found in extracts of CSF, 163 were found in plasma and 243 were found in brain tissue samples of Tg and control animals. Differences in metabolic profiles partially divided study groups in the PCA score plot models (Fig. 1). Explained variance was 34% for CSF samples, 48% for plasma samples and 62% for brain tissue samples. Repeatability of analyses is expressed by QC samples.

The data were evaluated by supervised univariate (box plots and *t* test) and multivariate statistics (OPLS-DA). From the OPLS-DA S-plot, the 20 most discriminating metabolites, sorted according to the absolute size of values from the *y*-axis are depicted together with fold changes and *p* values in Table 1. In CSF from Tg rats compared with controls, we observed increased levels of thymidine, citrate/isocitrate, and carnitine and decreased levels of myo-inositol, *S*-adenosylmethionine, and creatinine. In plasma samples, glutamine, proline, spermine, creatinine and docosahexaenoic acid were the most discriminating metabolites. Differences between brain tissues of Tg and control rats were found in levels of phosphorylated compounds (cytidine 5′-diphosphate, ATP, ADP, deoxyguanosine 5′-diphosphate, 3-phosphoglycerate) and degradation products of purine species (xanthine, hypoxanthine). OPLS-DA score scatterplots and S-plots for every biological material are shown in Additional file 1: Figures S2–S4.

### Untargeted metabolomic analysis

The samples of rats with tauopathy and controls were subjected to untargeted metabolomic analysis. A total of 390 features were found in extracts of CSF, 762 were found in plasma and 422 were found in brain tissue samples of Tg and control animals. PCA score plots with explained variance of 42% for CSF samples and 37% for plasma and brain tissue samples revealed group separation only in CSF and plasma samples (Fig. 1). Because almost no differences in PCA of brain extracts were found, obtained features were not subjected to structure identification.

Discriminating features of CSF and plasma samples between rats with tauopathy and controls from OPLS-DA S-plots are depicted in Table 2. Our results show changes in phosphatidylcholines (PCs) mainly with C16 and C18 side chains differing in the degree of saturation. Most discriminating PCs from CSF were elevated in tauopathy-affected rats compared with controls. However, four identified lipids [PC(20:2/0:0), LysoPC(20:3), PC(20:0/0:0), LysoPC(22:5)] were decreased in the plasma samples. OPLS-DA score scatterplots and S-plots of every biological material are presented in Additional file 1: Figures S5–S7 as well as in supplementary information about most discriminant features from untargeted analysis of brain tissue samples (Additional file 1: Table S4, Figure S10).

### Confirmation study

**Fig. 2:**
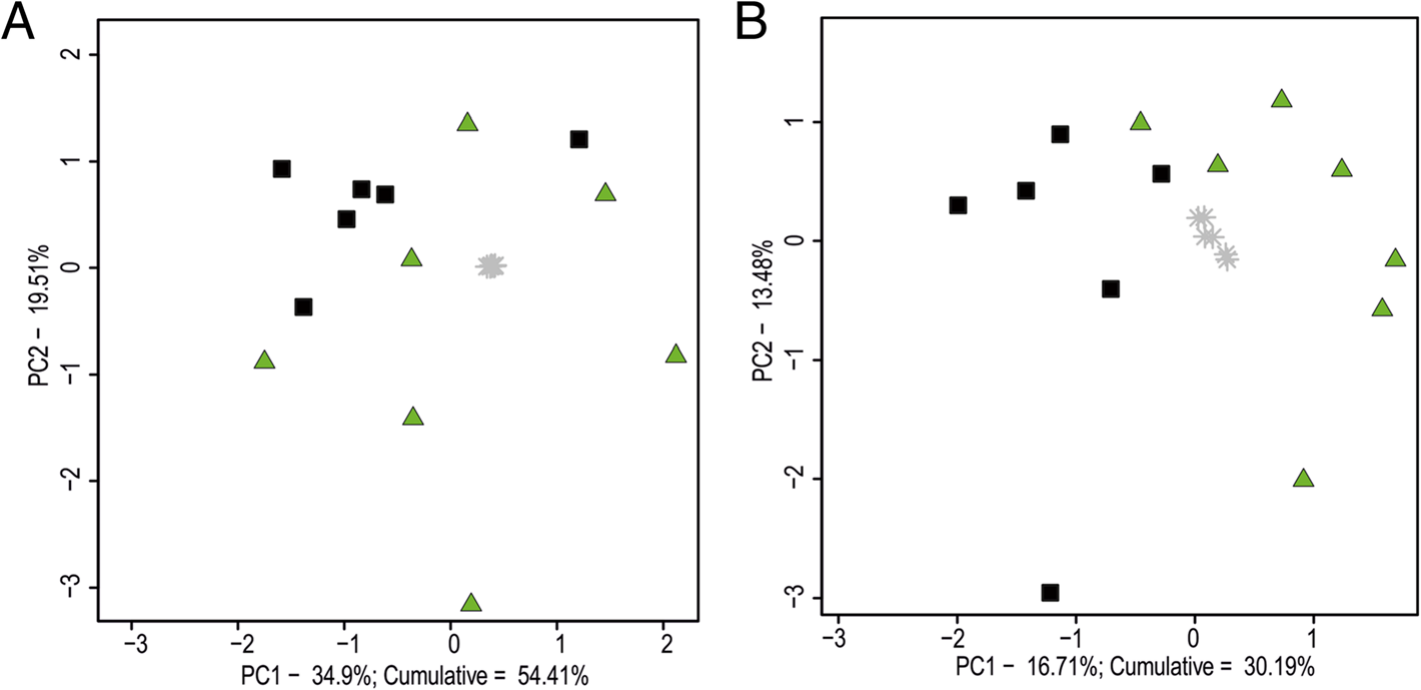
Confirmation study. Principal component analysis score plots of cerebrospinal fluid samples from transgenic rats (*green triangles*) and controls (*black squares*). **a** Targeted analysis. **b** Untargeted analysis

**Fig. 3:**
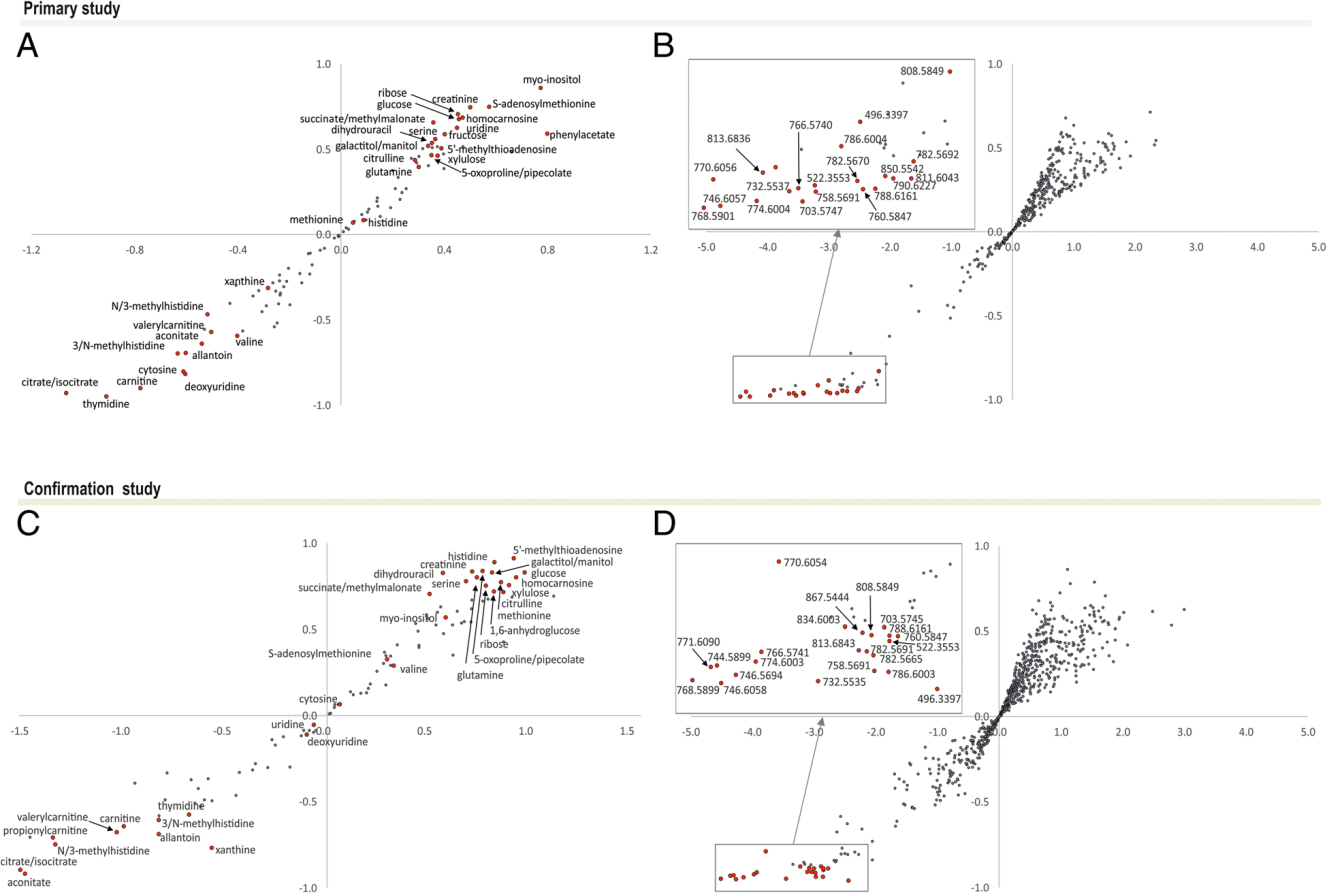
Orthogonal projections to latent structures discriminant analysis (OPLS-DA) S-plots of targeted (**a**, **c**) and untargeted (**b**, **d**) metabolomics from primary and confirmation study of cerebrospinal fluid samples. Twenty most discriminating metabolites/features from OPLS-DA analyses that were found in studies are highlighted (*red dots*)

CSF samples from SHR72 rats and controls were subjected to confirmation measurement by targeted and untargeted metabolomic analysis. The samples were obtained independently of the preliminary study (from other animals at different times). Unsupervised PCA clearly separated Tg and control rats with explained variance of 54% for targeted analysis and 30% for untargeted analysis (Fig. 2). Data from OPLS-DA analysis are shown in Additional file 1: Figures S8 and S9 and Tables S2 and S3. A detailed comparison of 20 of the most discriminating metabolites/features from OPLS-DA in the primary study is depicted in Fig. 3. As shown, most of them were found to be significant in both of the analyses.

## Discussion

Progressive neurofibrillary pathology in Tg rats was induced by expression of truncated tau, which consists of four microtubule-binding domains (4R) and a proline-rich region (151–391/4R). Neurofibrillary degeneration in these animals displays biochemical and histopathological features similar to those of human tau neurodegeneration, such as extensive formation of detergent-insoluble tau protein complexes and thioflavin S reactivity.

**Fig. 4:**
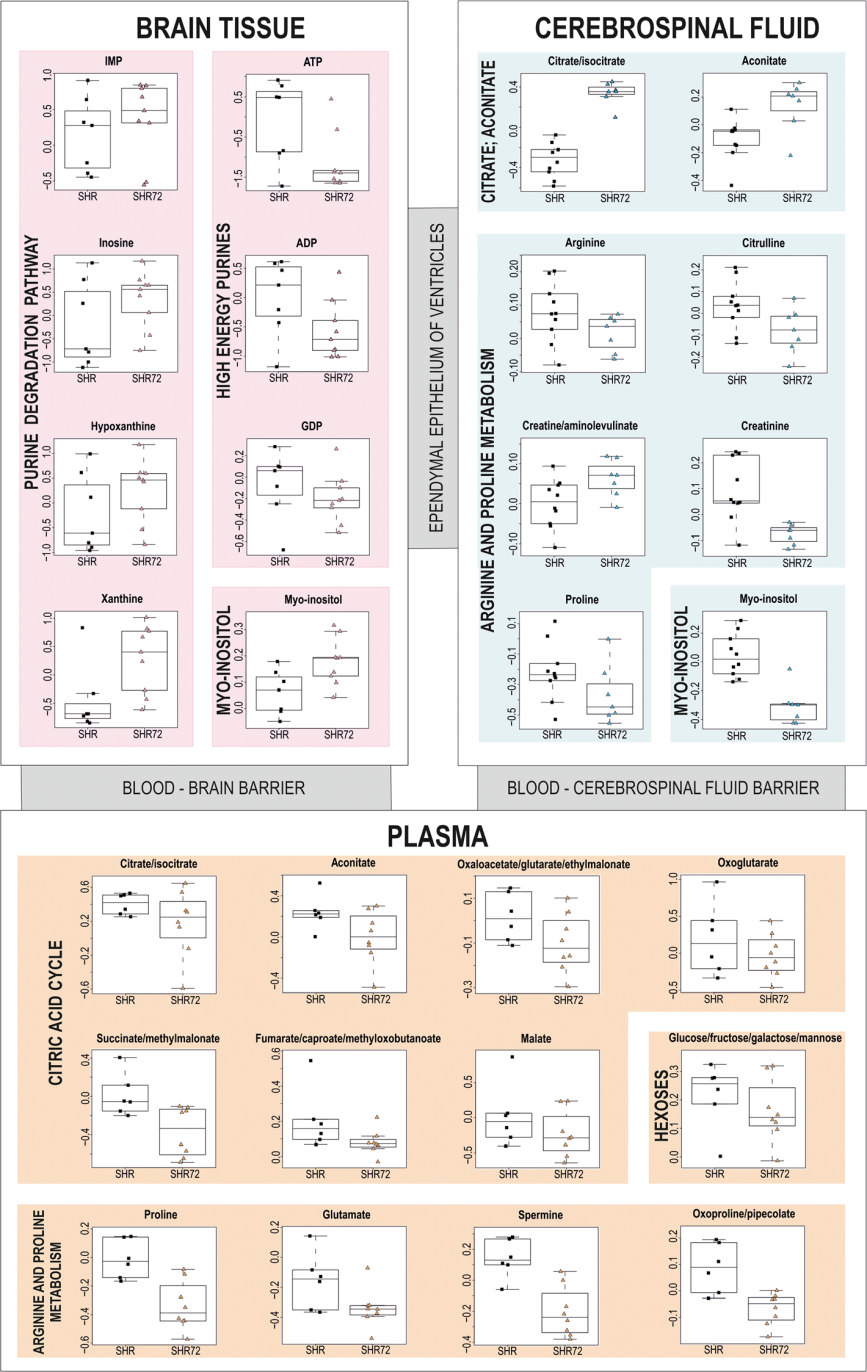
Boxplots of selected metabolites found in cerebrospinal fluid, plasma and brain tissue samples of transgenic rats (SHR72) and controls (SHR). *ADP* Adenosine 5′-diphosphate, *ATP* Adenosine 5′-triphosphate, *GDP* Guanosine 5´- diphosphate

Targeted and untargeted metabolomic analyses were used to identify metabolic pathways affected by the neurodegenerative process. Interesting differences in the levels of metabolites in CSF and plasma were observed (Fig. 4). Both biological liquids represent an acceptable source for metabolomic analyses, with CSF being the ‘gold standard’ in current AD biomarker studies. Moreover, the metabolic profile of CSF closely reflects brain-specific changes. Its composition is regulated by a system of barriers forming interfaces between blood, brain tissue and CSF.

### Statistical analysis

Metabolomic statistical analysis requires complex data evaluation using both univariate and multivariate methods. In univariate statistics, the *t* test is predominantly used; however, its results are limited by the difficulty of evaluation and comparison between studies as a result of the strong dependence on the number of samples in groups, normality and the presence of outlying values. In univariate statistics, box plots and calculation of *p* values from parametric two-sample and two-tailed *t* tests were used for our data where more than 85% metabolites were normally distributed (Shapiro-Wilk test).

In our study a limited number of samples per group (*n* = 10 for the primary study and *n* = 6 for the confirmation study) was selected for ethical reasons. The power of the study was calculated to be 0.29 and 0.48 for six and ten samples, respectively, using ∆ = 0.9, SD = 1 and significance level = 0.05. Many papers with limited numbers of samples for various reasons (e.g., rare disorders, expensiveness, inaccessibility, ethical reasons) have been published in recent years, in both the field of metabolomics and elsewhere. However, only a limited number of papers consider the power of the study, which represents the uncertainty and potential false negativity of the statistical tests used. The lower power of our study may be due to the differences between the lists of the most discriminating metabolites in the primary and confirmation studies.

The way to reduce potential false positivity in multidimensional data is to apply the Bonferroni correction to the results of the *t* test. This correction is conservative in the sense that, although it reduces the number of false-positive results, it also reduces the number of true discoveries. Therefore, it is recommended that it be used more as a guide than as a strict rule. Our data indicate more significant changes in CSF than with plasma and the brain in both targeted and untargeted metabolomic analyses (*see t* test results in Tables 1 and 2).

In metabolomics, univariate and multivariate statistical tests should be performed together. PCA as an unsupervised method is the most important in the determination of general behaviour and clustering of samples without initial knowledge of data grouping, making PCA a crucial part of metabolomic statistical analysis. Supervised PLS-DA (or its orthogonal projection OPLS-DA) is used to determine the most discriminant metabolites differentiating the groups. However, there is a high risk of false-positive results when this is used alone without interpreting the results in conjunction with univariate and unsupervised multivariate methods. The better the agreement between these three statistical approaches, the higher the validity of the study.

Additionally, to confirm the results from a primary metabolomic study and fortify the conclusions, an independent study should be performed. Even a small confirmative study brings a significant improvement in the certainty of the most discriminant metabolites, the potential biomarkers. The results from OPLS-DA, boxplots and the *t* test (Tables 1 and 2 and Figs. 3, 4, 6 and 7) show better agreement of discriminating metabolites in untargeted metabolomics than targeted metabolomics of CSF because of the different metabolite classes (lipids) detected with lower *p* values.

### Targeted metabolomics of cerebrospinal fluid and plasma

Significantly elevated levels of citrate/isocitrate and aconitate were detected in the CSF of Tg rats compared with the controls. Physiologically, the concentration of citrate in CSF is present in substantially higher levels than in plasma [26]. This concentration imbalance is attributed to citrate released from astrocytes [27]. The activation of astrocytes and microglial cells may therefore explain the difference in citrate levels between CSF of Tg rats and CSF of controls. Glial activation was reported in a Tg tauopathy model [28] as well as in AD [29]. In contrast to CSF findings, decreased levels of all citric acid cycle intermediates (oxaloacetate, citrate/isocitrate, aconitate, oxoglutarate, succinate, fumarate and malate) were found in plasma of Tg rats (Fig. 4). The citric acid cycle with oxidative phosphorylation is the main metabolic pathway for ATP production. In tauopathy rats, reduced citric acid cycle intermediates and glucose level (entering to glycolysis) pointed to impaired energy metabolism.

**Fig. 5:**
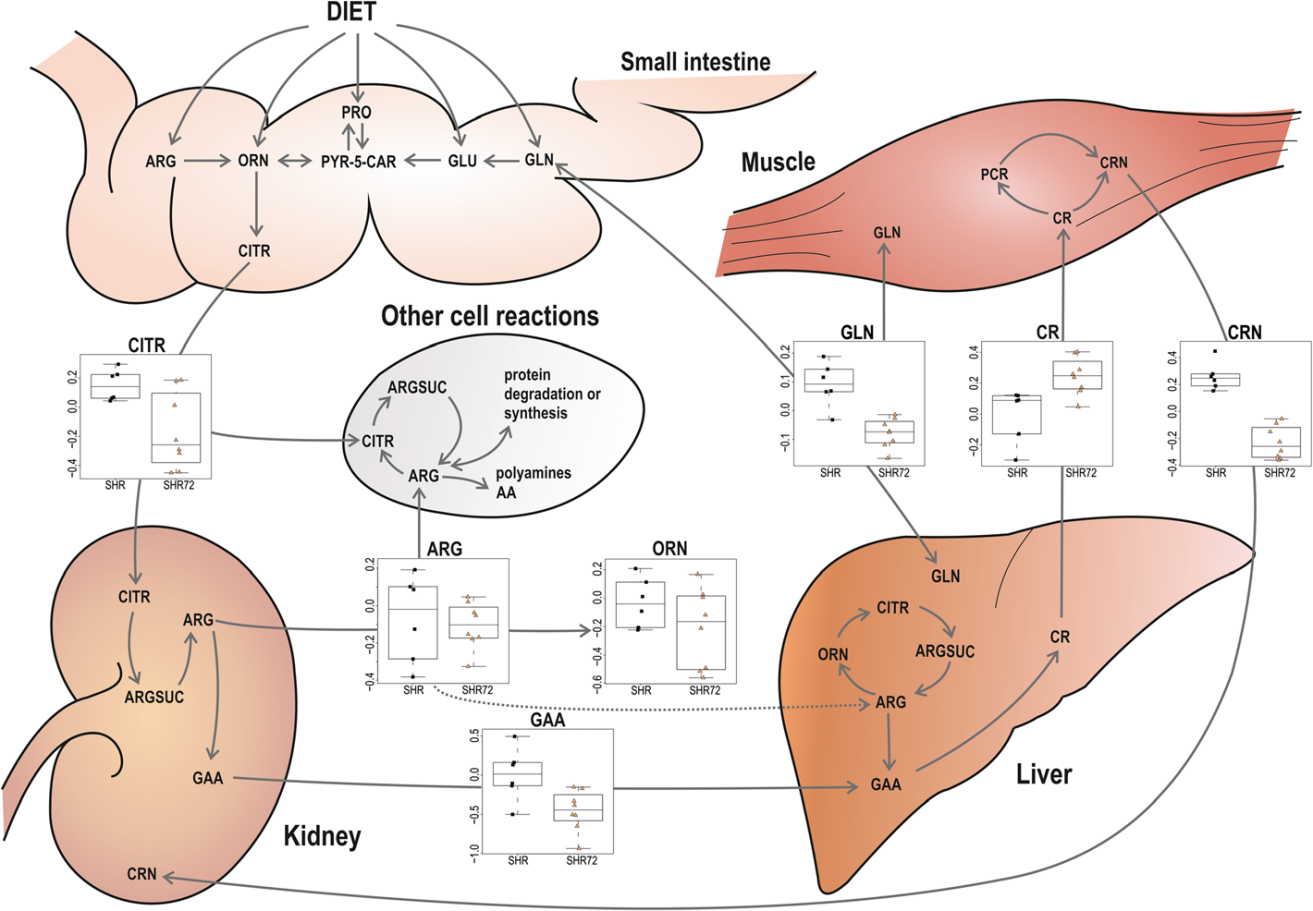
Changes in arginine metabolism that were found in plasma of transgenic rats (SHR72) and controls (SHR). *AA* Amino acids, *ARG* Arginine, *ARGSUC* Argininosuccinate, *CITR* Citrulline, *CR* Creatine, *CRN* Creatinine, *GAA* Guanidinoacetate, *GLN* Glutamine, *GLU* Glutamate, *ORN* Ornithine, *PCR* Phosphocreatine, *PRO* Proline, *PYR-5-CAR* Pyrroline-5-carboxylate

Changes in arginine and proline metabolism were observed in rats with tauopathy compared with controls. Multiple organ cooperation (e.g., kidney, small intestine, liver), different subcellular localisation and expression levels of enzymes involved in arginine metabolism complicate the biological interpretation. Our results revealed decreased levels of arginine, proline, oxoproline and creatinine in CSF and plasma samples of Tg rats. Moreover, plasma levels of guanidinoacetate, ornithine, glutamine, glutamate and spermine were also reduced. On the contrary, only creatine levels in both matrices were increased (Figs. 4 and 5). Homeostasis of arginine concentration in plasma depends on endogenous biosynthesis and catabolism, protein turnover and dietary intake. Approximately 60% of net arginine synthesis in adult mammals is produced from citrulline in the kidneys [30]. Decreased levels of citrulline in affected animals may be explained by impaired citrulline biosynthesis or by its elevated use in arginine metabolism. Arginine is an important precursor of many compounds (e.g., creatine, nitric oxide, polyamines, agmatine). A large amount of arginine is metabolised to creatine via inter-organ cooperation of the kidney, pancreas and liver [30]. Creatine is then transported by the blood and taken up by muscle cells. In our results, only creatine level was increased in arginine catabolism. Creatine has an anti-oxidative function, reduces inflammatory responses and plays an important role in energy metabolism via the creatine/phosphocreatine system [31, 32]. Creatinine is produced by irreversible and spontaneous conversion of creatine and creatine phosphate. Although the formation of creatinine is reasonably constant, it can depend on age, sex, ethnicity, dietary protein intake, renal function and total muscle mass [33, 34]. A similar metabolic profile (increased creatine concentration in plasma or urine and decreased urinary creatinine excretion) was described previously for some (neuro)muscular disorders [35] and vitamin E-deficient animals [36]. The reduction of skeletal muscles, increased membrane fragility and reduced creatine concentration in the remaining muscle mass are often attributed to this observation. Moreover, Loike et al. [37] showed that elevated extracellular creatine concentration downregulates its transport to myoblasts or myotubes. A decreased level of vitamin E was reported in plasma of patients with AD [38].

Increased levels of 3-methylhistidine/*N*-methylhistidine were found in the CSF and plasma of tauopathy animals (Table 1). Methylhistidine is formed by post-translational methylation of histidine residues in actin and myosin, and its excretion is therefore associated with muscular protein degradation [39]. Although the variation in weight among controls and Tg rats was not higher than 5%, elevated plasma level of methylhistidine may indicate increased myofibrillar protein catabolism.

### Untargeted metabolomics of CSF and plasma

**Fig. 6:**
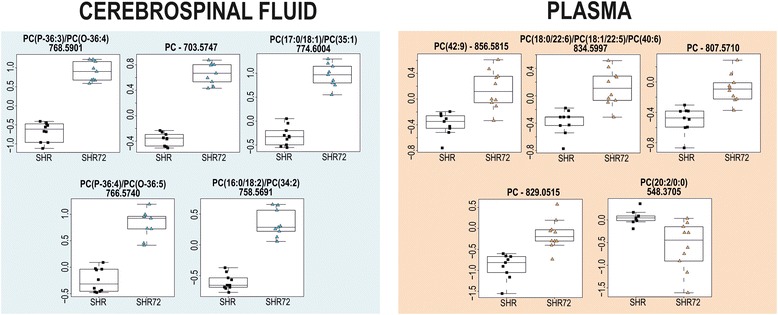
Box plots of discriminating features found in cerebrospinal fluid and plasma of transgenic rats (SHR72) and controls (SHR) by untargeted metabolomic analysis

Differences in PC levels in CSF and plasma samples of Tg rats and controls were found by untargeted metabolomic analysis (Fig. 6). PCs are a class of glycerophospholipids that are essential as components of the cell membrane and in cellular signalling. Various lengths and saturation degrees of fatty acids are typical of the structure of PCs [40]. Altered PC metabolism has been observed in AD [41, 42]. A significant decrease in the levels of PC(16:0/20:5), PC(16:0/22:6) and PC(18:0/22:6) was reported in the plasma of patients with AD [43]. One possible explanation for this observation could be phospholipase dysfunction that accompanies AD pathology [44, 45]. Orešič et al. [41] also published an observation of a slight decrease of some PCs concentrations [PC(16:0/18:2) PC(O-18:0/18:2) and PC(18:0/20:4)] in the blood of subjects with AD. This is in contrast with our data showing elevated levels of most PCs in both matrices. The increased membrane breakdown caused by neurodegeneration may be a possible explanation. It should be considered that our samples were not from human patients with AD, but from Tg rats expressing human tau protein. In addition, more important changes were found in CSF than in plasma, which will be subject of a following study.

### Targeted metabolomics of brain tissue

Analysis of brain tissue was also performed. No group separation of Tg and control rats was observed in the PCA, although all Tg rats had the same levels of insoluble tau. The brain microdissection procedure likely accounted for this observation. However, trends in levels of some metabolites (described below) apparent in other statistical methods (box plots, OPLS-DA) were visible.

Altered purine metabolism associated with neurodegenerative processes has been described in AD [46, 47]. Decreased levels of energy-rich diphosphates and triphosphates (ATP, ADP, inosine 5′-diphosphate, guanosine 5′-diphosphate) and increased levels of purine bases (hypoxanthine, inosine, xanthine, xanthosine) were found in the brains of Tg animals compared with controls (Fig. 4). This observation suggests the elevated purine catabolism associated with energy deprivation in the affected tissues. The source of these purine bases could also be DNA breakdown resulting from neural apoptosis. Our results are in agreement with those of a previous study that showed an elevated fold change of purine bases in a APP/PS1 Tg mice model vs. controls [48].

A decreased level of phosphocreatine generated from ATP and creatine by creatine kinase also points to impaired energy metabolism in brain tissues of Tg rats. An ATP shortage in Tg animals probably causes its reduced synthesis. This is in accord with human data from mildly demented subjects with AD [49].

Myo-inositol is a biomarker that is currently used for the diagnosis of MCI and AD in humans [50]. An elevated level of this compound was found in brain tissue samples of Tg rats compared with controls (Fig. 4). As previously described, an increased level of myo-inositol can reflect glial activation or proliferation which accompanies neuronal dysfunction or loss [51]. Elevated consumption of myo-inositol by brain glial cells might cause its decreased level in the CSF of Tg animals (Fig. 4).

### Confirmatory metabolomic study of CSF

**Fig. 7:**
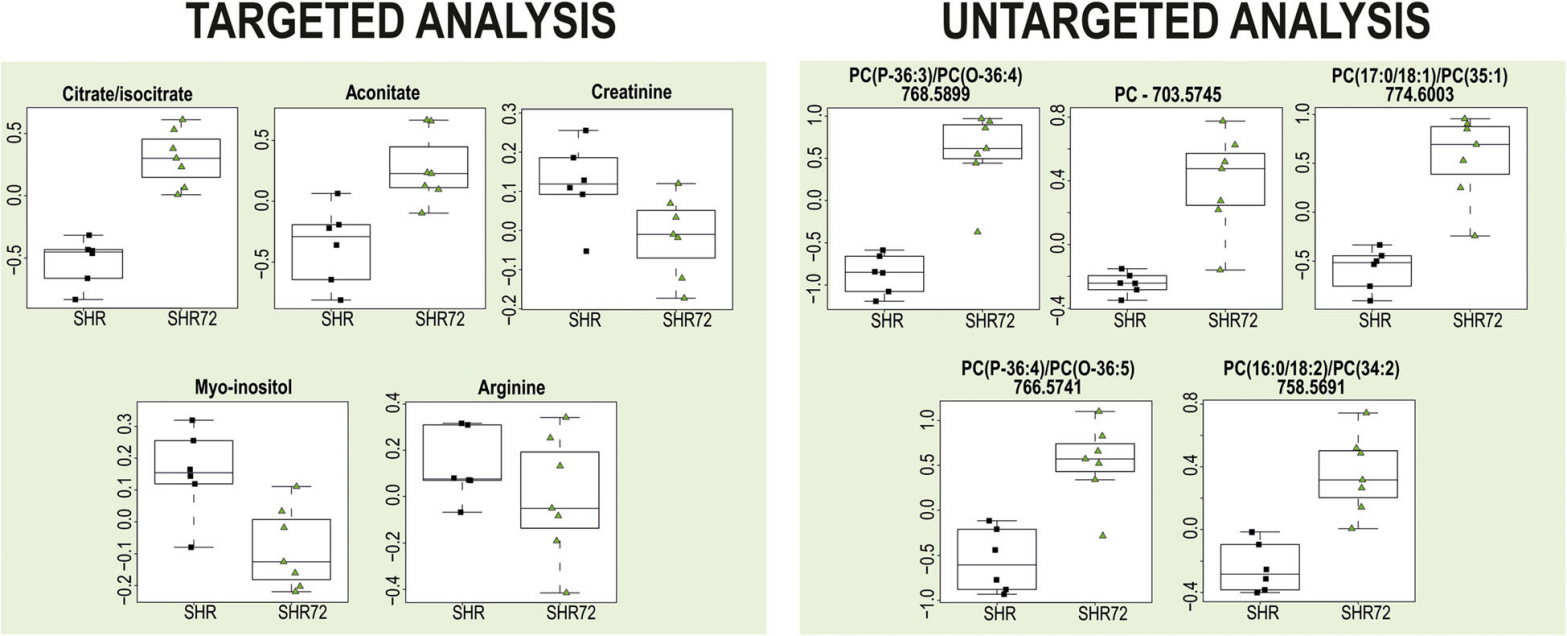
Confirmation of CSF results with primary study. Boxplots of discriminating metabolites/features that were found in CSF of transgenic rats (SHR72) and controls (SHR) by targeted and untargeted metabolomic analysis

To confirm the above-described results, an independent study of CSF from rats with tauopathy was performed. Similar trends in levels of discriminating metabolites/features were found in the samples collected from another group of Tg animals and controls (Figs. 3 and 7). In the non-targeted metabolomic analysis, where statistically more significant differences were observed, 10 of the 20 most discriminating metabolites were present in both studies (based on OPLS-DA results). In targeted metabolomic analysis, 7 of the top 20 metabolites were present in both studies. Citrate together with aconitate and creatinine in targeted metabolomic analysis, as well as characteristic PCs in untargeted metabolomic analysis, were found to be the most distinguishing compounds. These compounds may be considered as potential biomarkers of the tauopathy process in CSF. However, observed discrepancies caused by low power of primary and confirmatory metabolomic studies indicate a limitation of the approach. Therefore, the metabolites with low statistical significance should not be overestimated and considered as biomarkers, as described in the Statistical analysis of discussion section above.

## Conclusions

We have identified several metabolic changes affected by the tauopathy process that may be considered as potential markers for diagnosis of tauopathies in humans. Moreover, some of the identified pathways have previously been reported to be connected to neurodegeneration. We thus believe that detailed understanding of molecular processes behind the changes can lead to development of specific analytical methods and ultimately to monitoring of these features in ongoing anti-tau clinical trials.

## Additional file

Additional file 1: Table S1. Abbreviations of metabolites used in targeted metabolomic analysis. **Tables S2**, **S3.** Results of analysis of CSF from confirmatory study: targeted analysis (Table S2) and untargeted (Table S3). **Table S4.** Twenty most discriminating features from untargeted analysis of brain tissue samples. **Figure S1.** Expression levels of insoluble tau proteins in brainstem of transgenic animals used for study. **Figures S2**, **S3**, **S4**, **S5**, **S6**, **S7.** OPLS-DA score scatterplots and S-plots built for targeted and untargeted metabolomics: CSF targeted (Fig. S2) and untargeted (Fig. S5) metabolomics, plasma targeted (Fig. S3) and untargeted (Fig. S6) metabolomics, brain tissue targeted (Fig. S4) and untargeted (Fig. S7) metabolomics. **Figures S8**, **S9.** OPLS-DA score scatterplots and S-plots built for targeted and untargeted metabolomics of CSF in confirmation study: targeted (Fig. S8) and untargeted (Fig. S9) metabolomics. **Figure S10.** Box plots of most discriminating features of brain tissue samples in untargeted metabolomic analysis. (DOCX 4660 kb)

## Abbreviations

AA: Amino acids
Aβ: Amyloid-beta
acMet: *N*-acetylmethionine
AD: Alzheimer’s disease
ADP: Adenosine 5′-diphosphate
APP: Amyloid precursor protein
ARG: Arginine
ARGSUC: Argininosuccinate
ATP: Adenosine 5′-triphosphate
BEH: Ethylene bridged hybrid
C5: Valerylcarnitine
C12: Dodecanoylcarnitine
C14: Tetradecanoylcarnitine
C14:1: Tetradecenoylcarnitine
C14:1OH: Hydroxytetradecenoylcarnitine
C16: Hexadecanoylcarnitine
C16:1OH: Hydroxyhexadecenoylcarnitine
C16OH: Hydroxyhexadecanoylcarnitine
C18:1: Octadecenoylcarnitine
CDP: Cytidine 5′-diphosphate
CITR: Citrulline
CR: Creatine
CRN: Creatinine
CSF: Cerebrospinal fluid
CV: Coefficient of variation
dGDP: Deoxyguanosine 5′-diphosphate
DHA: Docosahexaenoic acid
dimGly_nh2isobut: Dimethylglycine/ 2-aminoisobutyric acid
EDTA: Ethylenediaminetetraacetic acid
GAA: Guanidinoacetate
GLN: Glutamine
GLU: Glutamate
GuaBut: Guanidinobutanoate
hCar: Homocarnosine
Hx: Hypoxanthine
IDP: Inosine 5′-diphosphate
LC-MS: Liquid chromatography-mass spectrometry
MCI: Mild cognitive impairment
mHis_NmHis: 3-Methylhistidine/ *N*-methylhistidine
MRM: Multiple reaction monitoring
MSI: Metabolomics Standards Initiative
*m/z*: Mass-to-charge ratio
NAcGlcnh2_NAcGalnh2_NAcManh2: *N*-acetylglucosamine/ *N*-acetylgalactosamine/ *N*-acetylmannosamine
NADP: Nicotinamide adenine dinucleotide phosphate
NaMN: Nicotinamide mononucleotide
OPLS-DA: Orthogonal projections to latent structures discriminant analysis
oPro_pipec: 5-Oxoproline/ pipecolate
ORN: Ornithine
PC: Phosphatidylcholine
PCA: Principal component analysis
PCr: Phosphocreatine
3PG: Glycerate 3-phosphate
PRO: Proline
PYR-5-CAR: Pyrroline-5-carboxylate
QC: Quality control samples
rib5P_xyl5P: Ribose 5-phosphate/ xylulose 5-phosphate
S-ArMet: *S*-adenosylmethionine
SDS: Sodium dodecyl sulphate
SHR: Control rats
SHR72: Transgenic rats with tauopathy
Succ_mma: Succinate/ methylmalonate
Tg: Transgenic model
Tr: Thymidine
UDP: Uridine 5′-diphosphate
