# Supplementary material for: Metabolic status of CSF distinguishes rats with tauopathy from controls
Source: Alzheimers Res Ther. 2017 Sep 21;9:78. doi: 10.1186/s13195-017-0303-5 (PMC5609022; doi:10.1186/s13195-017-0303-5)
Supplement: Additional file 1: Table S1. — Abbreviations of metabolites used in targeted metabolomic analysis. Tables S2, S3. Results of analysis of CSF from confirmatory study: targeted analysis (Table S2) and untargeted (Table S3). Table S4. Twenty most discriminating features from untargeted analysis of brain tissue samples. Figure S1. Expression levels of insoluble tau proteins in brainstem of transgenic animals used for study. Figures S2, S3, S4, S5, S6, S7. OPLS-DA score scatterplots and S-plots built for targeted and untargeted metabolomics: CSF targeted (Fig. S2) and untargeted (Fig. S5) metabolomics, plasma targeted (Fig. S3) and untargeted (Fig. S6) metabolomics, brain tissue targeted (Fig. S4) and untargeted (Fig. S7) metabolomics. Figures S8, S9. OPLS-DA score scatterplots and S-plots built for targeted and untargeted metabolomics of CSF in confirmation study: targeted (Fig. S8) and untargeted (Fig. S9) metabolomics. Figure S10. Box plots of most discriminating features of brain tissue samples in untargeted metabolomic analysis. (DOCX 4660 kb) [file 13195_2017_303_MOESM1_ESM.docx]

**Supplementary material**

Table S1: Abbreviations of metabolites used in targeted metabolomic analysis

| **Full name of metabolite** | **Abbreviation of metabolite** |
| --- | --- |
| pyruvate | PYR |
| malonate  3-hydroxybutanoate | MALONATE  ohBUTANOATE |
| 2-hydroxyisobutyrate  2-hydroxybutyate  4-hydroxybutyrate | ohBUTYRIC  ISOohBUTYRIC |
| uracil | U |
| fumarate  caproic acid  3-methyl-2-oxobutanoate | FUM  CAPROATE  3m2oBUTANOATE |
| succinate  methylmalonate | SUCC  MMA |
| 2-hydroxyisovalerate | 2ohisoVALERATE |
| 4-hydroxybenzaldehyde | 4ohBENZALDEHYDE |
| taurine | TAU |
| 5-oxoproline | 5oPRO |
| glutaconate  ketoleucine  mevalonolactone  3-methyl-2-oxopentanoate | GLUTACONATE  ketoLEU  mevalonoLACTONE  oPENTANOATE |
| propionylglycine | propGly |
| N-acetylalanine | NAcALA |
| aspartate | ASP |
| phenylacetate | PHEAC |
| salicylate  4-hydroxybenzoate | SALICYLATE  4ohBENZOATE |
| ethanolamine phosphate | EtOHNH2P |
| 5-hydroxymethyluracil | 5ohmU |
| isobutyrylglycine  butyrylGly | ISObutGLY  butGLY |
| 2-oxoglutarate | oGLUTARATE |
| 2-hydroxyglutarate | ohGLUTARATE |
| ribose | RIB |
| hydrocinnamate | hCINNAMATE |
| xanthine | X |
| orotic acid | OA |
| succinylacetone | SAC |
| 2-methyl-butyrylglycine  isovalerylglycine | mbutGLY  ivalGLY |
| glycylaminobutyric acid | GLY-nh2BUTANOATE |
| 1,6-anhydroglucose | 16anhydroGLC |
| 7-methylguanine | 7mG |
| 1-methylxanthine | 1mX |
| 3-(3-hydroxyphenyl)propanoate | 3-ohphePROPANOATE |
| phenyllactate | PHELACT |
| phosphoenolpyruvate | PEP |
| 2-furoylglycine | furoylGly |
| glycerone phosphate | GLYCERONEP |
| glycerol 3-phosphate | GLYCEROL3P |
| arginine | ARG |
| indole-3-acetate | INDOLE3Ac |
| citrulline | CITRUL |
| glucose  fructose  galactose  mannose | GLC  FRU  GAL  MAN |
| homovanillate | hVANILATE |
| 3-phosphoglycerate | GLYCERATE3P |
| leucyl-glycine | LEUGLY |
| N-acetylglutamate | NacGLU |
| 5-hydroxyindoleacetate | ohINDOLAc |
| N-acetylmethionine | acMET |
| citrate  isocitrate | CIT  iCIT |
| erythrose 4-phosphate | ERY4P |
| N,N-dimethylarginine | NNdimARG |
| phenylpropionylglycine | phenylpropGly |
| deoxyribose 5-phosphate | dRIB5P |
| ribose 5-phosphate  xylulose 5-phosphate | RIB5P  XYL5P |
| suberylglycine | suberylGly |
| formylglycinamide ribonucleoside | FGAr |
| pseudouridine | pseuUR |
| uridine | UR |
| leucyl-leucine | LEULEU |
| glucosamine 6-phosphate | GLCnh2P |
| glucose 1-phosphate  galactose 1-phosphate  glucose 6-phosphate  fructose 6-phosphate | GLC1P  GAL1P  GLC6P  FRU6P |
| sorbitol 6-phosphate | SORBITOL6P |
| deoxyguanosine | dGr |
| adenosine | Ar |
| N-argininosuccinate | ARGSUCC |
| N-acetylglucosamine 6-phosphate | NacGLCnh26P |
| arachidonic acid | AA |
| cytidine 2',3'-cyclic phosphate | 2-3cCMP |
| deoxycytidine 5'-monophosphate | dCMP |
| deoxyuridine 5'-monophosphate | dUMP |
| sialic acid | SIALIC |
| deoxythymidine 5'-monophosphate | dTMP |
| cytidine 5'-monophosphate | CMP |
| uridine 5'-monophosphate | UMP |
| docosahexaenoic acid | DHA |
| deoxyadenosine 5'-monophosphate | dAMP |
| melibiose  sucrose  lactose  maltose | MEL  SUC  LAC  MAL |
| adenosine 5'-monophosphate | AMP |
| deoxyguanosine 5'-monophosphate | dGMP |
| inosine 5'-monophosphate | IMP |
| guanosine 5'-monophosphate | GMP |
| succinyladenosine | SAR |
| deoxycytidine 5'-diphosphate | dCDP |
| deoxythymidine 5'-diphosphate | dTDP |
| cytidine 5'-diphosphate | CDP |
| uridine 5'-diphosphate | UDP |
| deoxyadenosine 5'-diphosphate | dADP |
| thiamine diphosphate | THIAMINPP |
| adenosine 5'-diphosphate | ADP |
| deoxyguanosine 5'-diphosphate | dGDP |
| inosine 5'-diphosphate | IDP |
| guanosine 5'-diphosphate | GDP |
| adenylosuccinic acid | SAMP |
| deoxycytidine 5'-triphosphate | dCTP |
| deoxyuridine 5'-triphosphate | dUTP |
| deoxythymidine 5'-triphosphate | dTTP |
| cytidine 5'-triphosphate | CTP |
| uridine 5'-triphosphate | UTP |
| choline cytidine 5'-diphosphate | CDPCHOLINE |
| deoxyadenosine 5'-triphosphate | dATP |
| deoxyinosine 5'-triphosphate | dITP |
| adenosine 5'-triphosphate | ATP |
| deoxyguanosine 5'-triphosphate | dGTP |
| inosine 5'-triphosphate | ITP |
| guanosine 5'-triphosphate | GTP |
| adenosine diphosphate ribose | ADPRIB |
| galactose uridine diphosphate  glucose uridine diphosphate | UDPGAL  UDPGLC |
| uridine diphosphate glucuronate | UDP-glucuronate |
| guanosine diphosphate fucose | GDPFUC |
| guanosine diphosphate mannose | GDPMAN |
| uridine diphosphate N-acetylglucosamine | UDP-NAcGLCnh2 |
| triiodothyronine | IIITHYRONINE |
| nicotinamide adenine dinucleotide | NAD |
| nicotinamide adenine dinucleotide phosphate | NADP |
| glycine | GLY |
| alanine  sarcosine  beta-alanine | ALA  sarcosine  bALA |
| N,N-dimethylglycine  2-aminoisobutyric acid | dimGLY  nh2isobutanoate |
| 3-aminoisobutyrate | 3nh2ISOBUTANOATE |
| 4-aminobutanoate | 4nh2BUTANOATE |
| serine | SER |
| cytosine | C |
| dihydrouracil | dihydroU |
| proline | PRO |
| valine | VAL |
| guanidinoacetate | GuaAc |
| betaine | BET |
| threonine  homoserine | THR  hSER |
| cysteine | CYS |
| taurine | TAU |
| N-methylhistamine | NmHISTAMINE |
| thymine | T |
| dihydrothymine | dihydroT |
| 5-oxoproline  pipecolate | oPRO  PIPECOLATE |
| N-acetylputrescine | NAcPut |
| 5-aminolevulinate  creatine | 5nh2LEVULINATE  CREATINE |
| leucine  isoleucine  alloisoleucine  norleucine  hydroxyproline | LEU  ILEU  alLE  NorLeu  hPRO |
| leucine | LEU |
| isoleucine  alloisoleucine | ILEU  alLE |
| hydroxyproline | hPRO |
| 6-aminohexanoate | 6nh2HEXANOATE |
| ornithine | ORN |
| glycyl-glycine | GLYGLY |
| asparagine | ASN |
| adenine | A |
| homocysteine | hCYS |
| hypoxanthine | HX |
| 4-guanidinobutanoate | GuaBUTANOATE |
| glutamine | GLN |
| lysine | LYS |
| glycyl-alanine | GLYALA |
| glutamate | GLU |
| methionine | MET |
| guanine | G |
| histidine | HIS |
| tiglylglycine  3-methylcrotonylglycine | tiglylGLY  3mCROTONYLGLY |
| alanyl-alanine | ALAALA |
| 2-aminoadipate | 2nh2ADIPATE |
| carnitine | C0 |
| phenylalanine | PHE |
| N-methylhistidine  3-methylhistidine | NmHIS  3mHIS |
| hexanoylglycine | hexGLY |
| N-acetylornithine | acORN |
| glycyl-valine | GLYVAL |
| N-acetylaspartate | NAcASP |
| glucosamine | GLCnh2 |
| tyrosine | TYR |
| phenylserine | PHENYLSER |
| phosphoserine | OPSER |
| homoarginine | hARG |
| glycyl-leucine  glycyl-norleucine | GLYLEU  GlyNorleu |
| alanyl-valine  alanyl-norvaline | ALAVAL  AlaNorval |
| glycyl-glycyl-glycine | GLYGLYGLY |
| glycyl-aspartate | GLYASP |
| benzoyl-alanine | BENZOYLALA |
| alanyl-asparagine | ALASN |
| alanyl-glycyl-glycine | ALAGLYGLY |
| acetylcarnitine | C2 |
| tryptophan | TRP |
| phosphocreatine | PCREATINE |
| propenoylcarnitine | C3-1 |
| aminoimidazole riboside | Air |
| propionylcarnitine | C3 |
| N-acetylserotonin | NAcSerotonin |
| alanyl-methionine | ALAMET |
| 5-hydroxytryptophane | ohTRP |
| N-acetylgalactosamine  N-acetylglucosamine  N-acetylmannosamine | NAcGALnh2  NAcGLCnh2  NAcMANnh2 |
| glycyl-phenylalanine | GLYPHE |
| N-acetyltyrosine | NAcTYR |
| deoxyuridine | dUR |
| butenylcarnitine | C4-1 |
| butyrylcarnitine | C4 |
| hydroxypropionylcarnitine | C3OH |
| alanyl-phenylalanine | ALAPHE |
| glycyl-tyrosine | GLYTYR |
| beta-alanyl-N-methylhistidine | bALAmHIS |
| homocarnosine | hCARNOSINE |
| thymidine | Tr |
| cytidine | Cr |
| tiglylcarnitine | C5-1 |
| leucyl-glycyl-glycine | LEUGLYGLY |
| valerylcarnitine | C5 |
| glycyl-glycyl-glycyl-glycine | GLYGLYGLYGLY |
| malonylcarnitine  hydroxybutyrylcarnitine | C3DC  C4OH |
| 6-hydroxymelatonin | ohMELATONINE |
| glutamyl-cysteine | GLUCYS |
| deoxyadenosine | dAr |
| deoxyinosine | dHr |
| 7,8-dihydroneopterine | dihydroBIOPTERINE |
| hexenoylcarnitine | C6-1 |
| 5-aminoimidazole-4-carboxamide riboside | AICAr |
| hexanoylcarnitine | C6 |
| 5-amino-4-imidazolecarboxylic acid ribonucleoside | CAIr |
| glycyl-tryptophan | GLYTRP |
| methylmalonylcarnitine  3-hydroxyisovalerylcarnitine | C4DC  C5OH |
| adenosine | Ar |
| inosine | Hr |
| homocystine | hCYStine |
| glutarylcarnitine  hydroxyhexanoylcarnitine | C5DC  C6OH |
| guanosine | Gr |
| xanthosine | Xr |
| octenoylcarnitine | C8-1 |
| 5-formamidoimidazole-4-carboxamide riboside | FAICAr |
| octanoylcarnitine | C8 |
| methylglutarylcarnitine | C6DC |
| histidyl-histidine | HISHIS |
| leucyl-tyrosine | LEUTYR |
| 5'-methylthioadenosine | 5mthioAr |
| nonayl-L-carnitine | C9 |
| pimelylcarnitine  hydroxyoctanoylcarnitine | C7DC  C8OH |
| decadienylcarnitine | C10-2 |
| decenoylcarnitine | C10-1 |
| decanoylcarnitine | C10 |
| nicotinamide mononucleotide | NAMN |
| 5-aminoimidazole-4-carboxamide ribotide | AICAR |
| dodecenoylcarnitine | C12-1 |
| dodecanoylcarnitine | C12 |
| tetradecadienylcarnitine | C14-2 |
| tetradecenoylcarnitine | C14-1 |
| tetradecanoylcarnitine | C14 |
| hydroxytetradecadienylcarnitine | C14-2OH |
| S-adenosylhomocysteine | SArhCYS |
| hydroxytetradecenoylcarnitine | C14-1OH |
| hexadecadienylcarnitine | C16-2 |
| hexadecenoylcarnitine | C16-1 |
| S-adenosylmethionine | sArMET |
| hexadecanoylcarnitine | C16 |
| hydroxyhexadecadienylcarnitine | C16-2-OH |
| hydroxyhexadecenoylcarnitine | C16-1OH |
| hydroxyhexadecanoylcarnitine | C16OH |
| octadecatrienylcarnitine | C18-3 |
| octadecadienylcarnitine | C18-2 |
| octadecenoylcarnitine | C18-1 |
| octadecanoylcarnitine | C18 |
| hydroxyoctadecenoylcarnitine | C18-1OH |
| eicosenoylcarnitine | C20-1 |
| 5'-phosphoribosyl-4-(N-succinocarboxamide)-5-aminoimidazol | SAICAR |
| eicosanoylcarnitine | C20 |
| 5-methyltetrahydrofolate | 5mTHF |
| 5-formyltetrahydrofolate | 5formylTHF |
| glutathione disulfide | GLUTATHIONESS |
| cytidine monophosphate N-acetylneuraminate | CMPNAcNEURAMINATE |
| coenzyme A | CoA |
| flavin adenine dinucleotide | FAD |
| acetyl coenzyme A | AcCoA |

Table S2: Targeted analysis of CSF – confirmation study. Twenty most discriminating metabolites from OPLS-DA analysis (sorted by absolute value of pcorr1 axis – variation related to variable magnitude). P-value, fold change and corrected α value (after Bonferroni correction) for these metabolites are shown.

| name | p1 | pcorr1 | fold change | t-test  p value  (α =4.95E-04) ^‡^ |
| --- | --- | --- | --- | --- |
| aconitate | -1.49 | -0.92 | 0.59 | 3.75E-03 |
| 5-methylthioadenosine | 0.92 | 0.91 | 1.21 | 3.00E-03 |
| citrate  isocitrate | -1.51 | -0.89 | 0.47 | 1.64E-05 |
| histidine | 0.83 | 0.89 | 1.25 | 3.13E-03 |
| 5-oxoproline  pipecolate | 0.77 | 0.84 | 1.08 | 4.62E-02 |
| creatinine | 0.72 | 0.84 | 1.14 | 4.02E-02 |
| glucose  fructose  galactose  mannose | 0.98 | 0.83 | 1.15 | 1.42E-01 |
| galactitol  manitol | 0.82 | 0.83 | 1.13 | 1.37E-01 |
| dihydrouracil | 0.57 | 0.83 | 1.12 | 1.33E-02 |
| homocarnosine | 0.93 | 0.80 | 1.21 | 3.59E-02 |
| glutamine | 0.74 | 0.80 | 1.12 | 3.42E-02 |
| serine | 0.69 | 0.78 | 1.23 | 1.30E-02 |
| methionine | 0.86 | 0.78 | 1.34 | 1.11E-01 |
| xanthine | -0.57 | -0.77 | 0.88 | 5.40E-03 |
| xylulose | 0.90 | 0.76 | 1.30 | 1.58E-01 |
| ribose | 0.79 | 0.75 | 1.20 | 2.56E-01 |
| N-methylhistidine  3-methylhistidine | -1.34 | -0.75 | 0.58 | 1.60E-02 |
| 1,6-anhydroglucose | 0.82 | 0.72 | 1.09 | 2.99E-01 |
| citrulline | 0.87 | 0.72 | 1.02 | 1.51E-01 |
| C3 | -1.36 | -0.71 | 0.79 | 2.70E-01 |

Table S3: Untargeted analysis of CSF – confirmation study. Twenty the most discriminating features from OPLS-DA analysis (sorted by absolute value of pcorr1 axis – variation related to variable magnitude). P-value, fold change and corrected α value (after Bonferroni correction) for these features are shown.

| *m/z* | p1 | pcorr1 | fold change | t-test  p value  (α =6.93E-05) ^‡^ |
| --- | --- | --- | --- | --- |
|  |  |  |  |  |
| 496.3397 | -2.44 | -0.96 | 0.66 | 1.41E-04 |
| 746.6058 | -4.27 | -0.95 | 0.27 | 4.68E-05 |
| 732.5535 | -3.45 | -0.95 | 0.43 | 8.37E-05 |
| 768.5899 | -4.52 | -0.95 | 0.23 | 4.57E-05 |
| 746.5694 | -4.15 | -0.94 | 0.28 | 5.03E-05 |
| 786.6003 | -2.86 | -0.94 | 0.59 | 7.88E-04 |
| 758.5691 | -2.98 | -0.93 | 0.55 | 3.54E-04 |
| 771.6090 | -4.36 | -0.93 | 0.21 | 6.61E-05 |
| 744.5899 | -4.31 | -0.93 | 0.26 | 9.12E-05 |
| 774.6003 | -3.98 | -0.92 | 0.30 | 1.63E-04 |
| 782.5665 | -2.99 | -0.91 | 0.56 | 1.58E-03 |
| 766.5741 | -3.93 | -0.91 | 0.31 | 3.98E-04 |
| 782.5691 | -3.04 | -0.91 | 0.50 | 2.98E-03 |
| 813.6843 | -3.11 | -0.91 | 0.43 | 3.35E-04 |
| 522.3553 | -2.85 | -0.89 | 0.54 | 3.35E-04 |
| 760.5847 | -2.78 | -0.89 | 0.66 | 5.19E-03 |
| 788.6161 | -2.85 | -0.89 | 0.54 | 4.42E-03 |
| 808.5849 | -3.00 | -0.89 | 0.50 | 3.81E-03 |
| 867.5444 | -3.08 | -0.88 | 0.56 | 5.71E-03 |
| 703.5745 | -2.89 | -0.88 | 0.49 | 7.77E-03 |


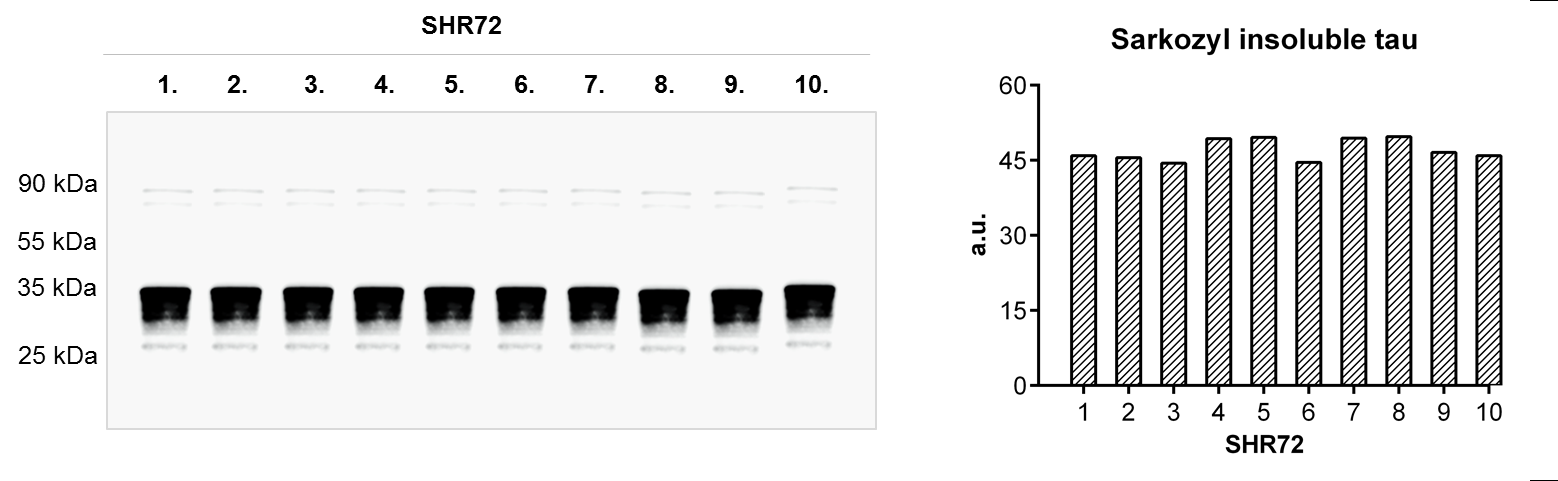


Figure S1: Expression levels of insoluble tau proteins in brain stem of transgenic animals.


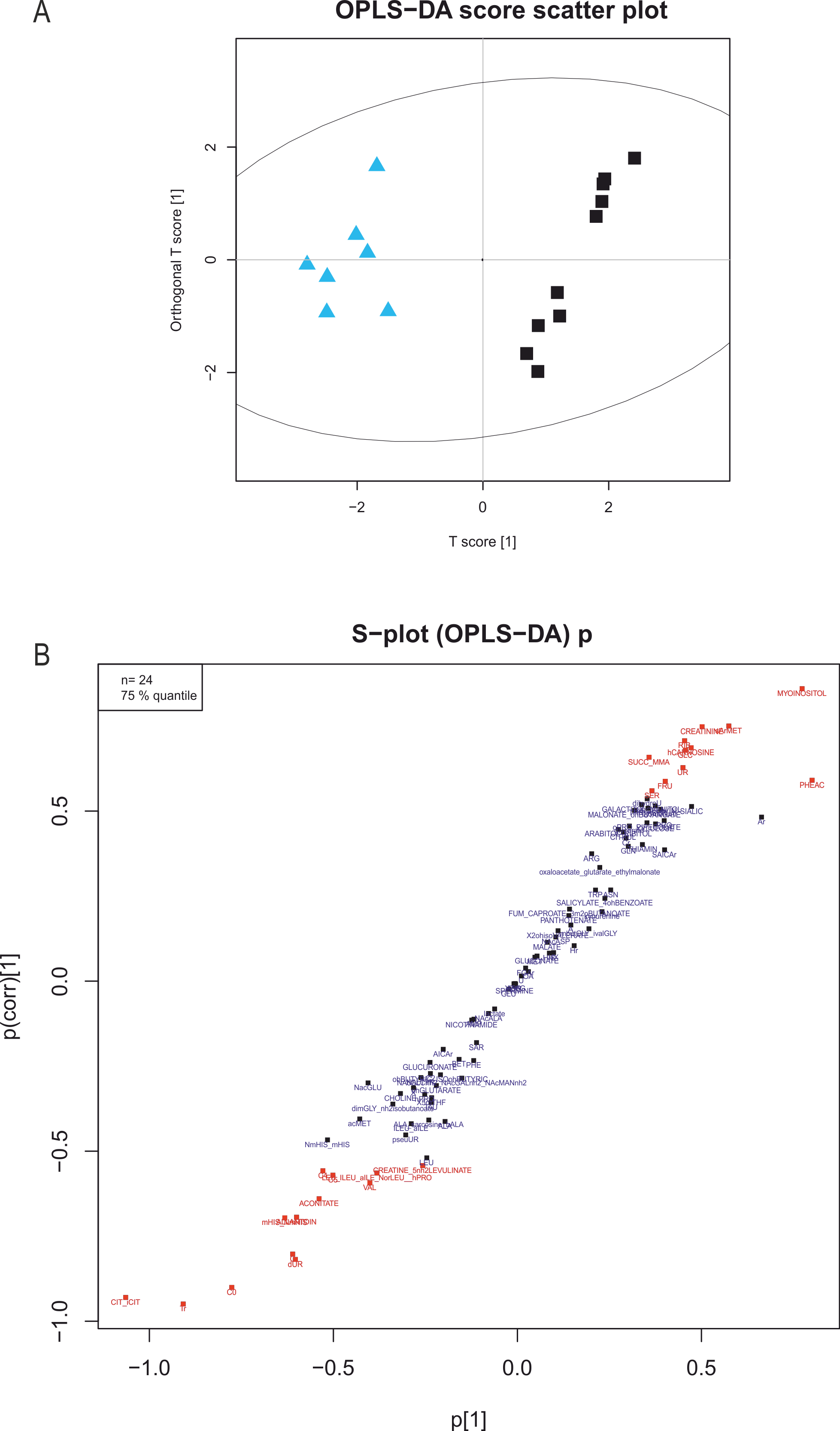


Figure S2: (A) OPLS-DA score scatter plot and (B) S-plot build for targeted metabolomic analysis of CSF samples (transgenic rats - blue triangles; controls - black squares).


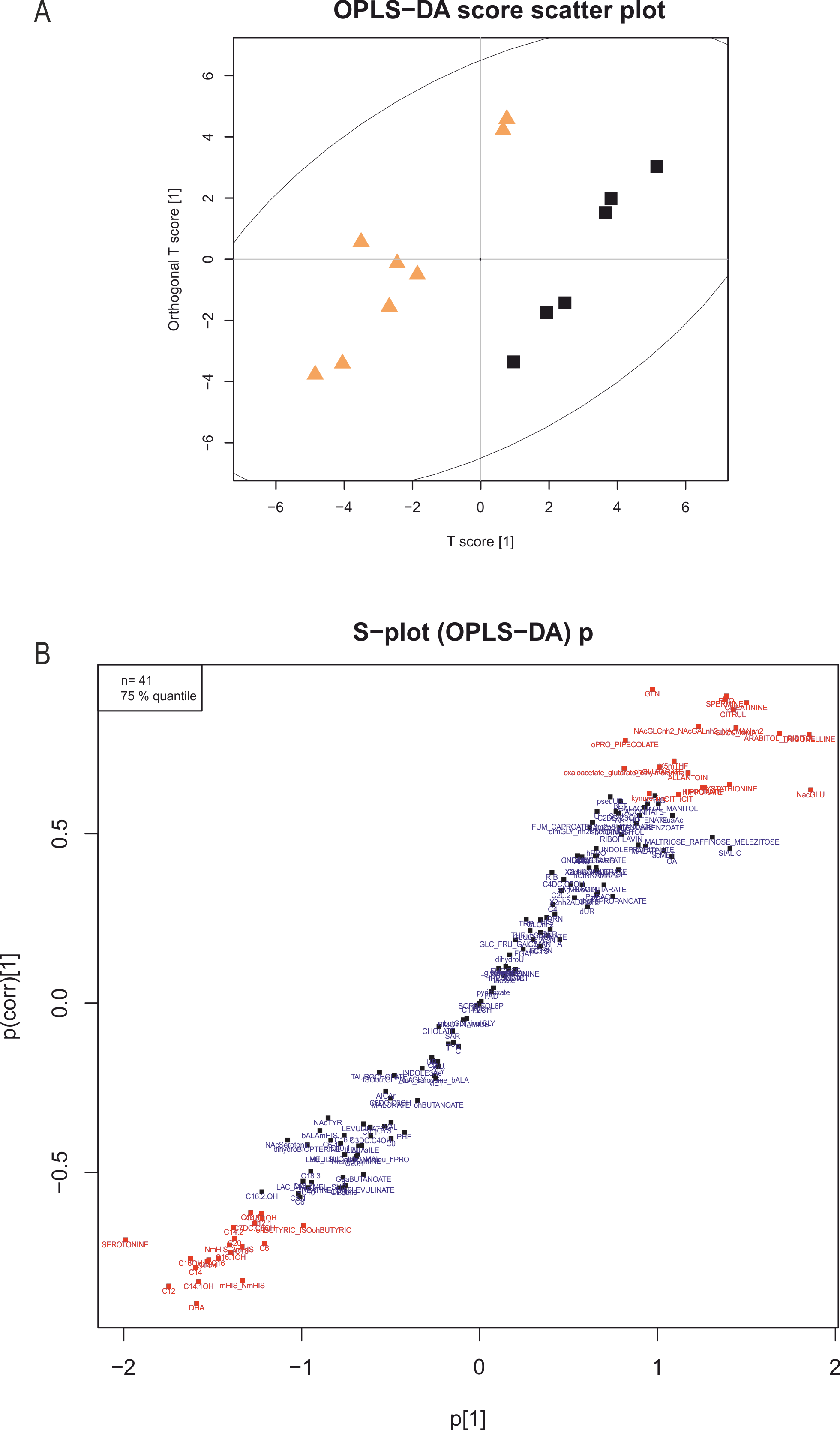


Figure S3: (A) OPLS-DA score scatter plot and (B) S-plot build for targeted metabolomic analysis of plasma samples (transgenic rats - orange triangles; controls - black squares).


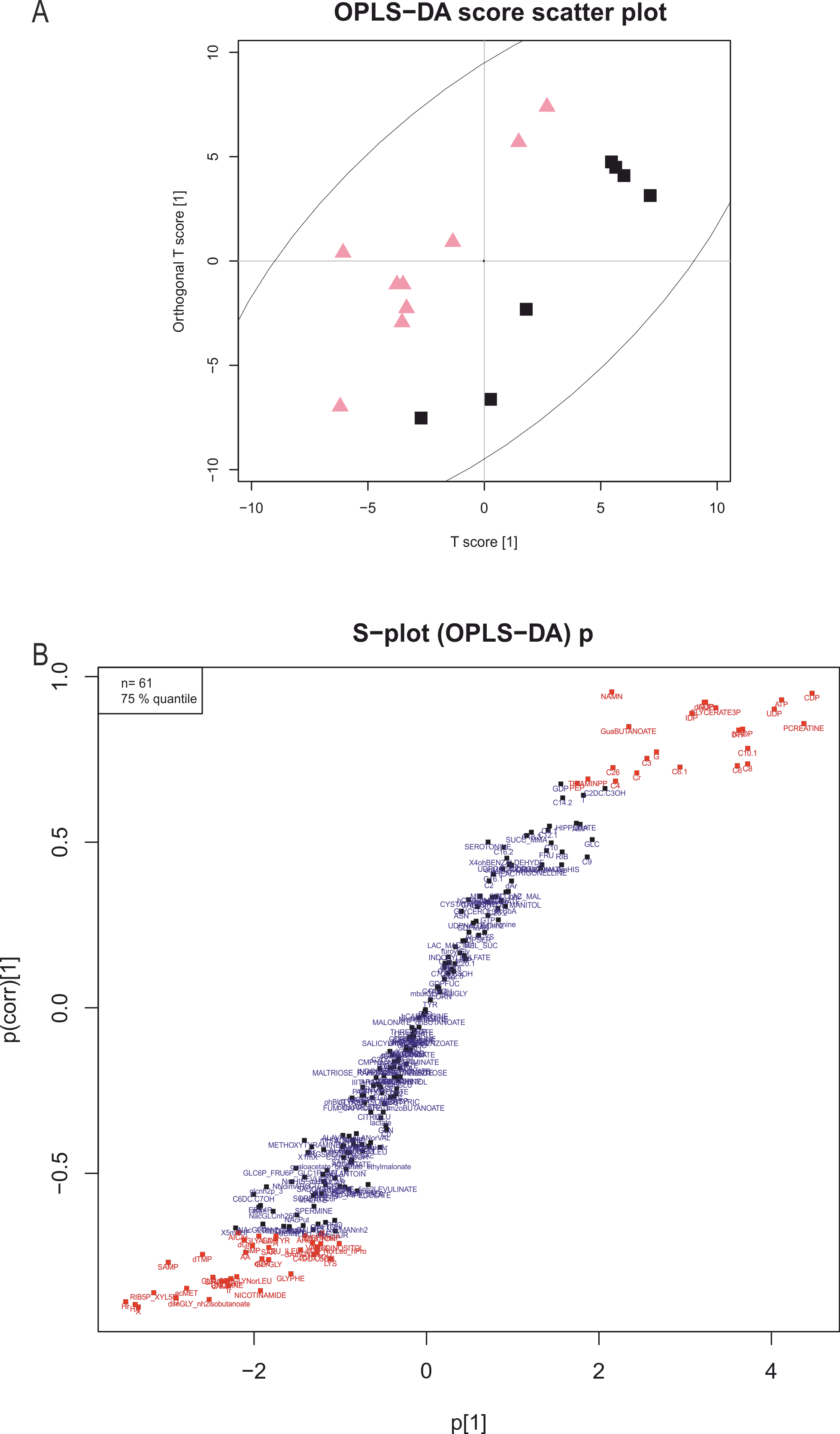


Figure S4: (A) OPLS-DA score scatter plot and (B) S-plot build for targeted metabolomic analysis of brain samples (transgenic rats - pink triangles; controls - black squares).


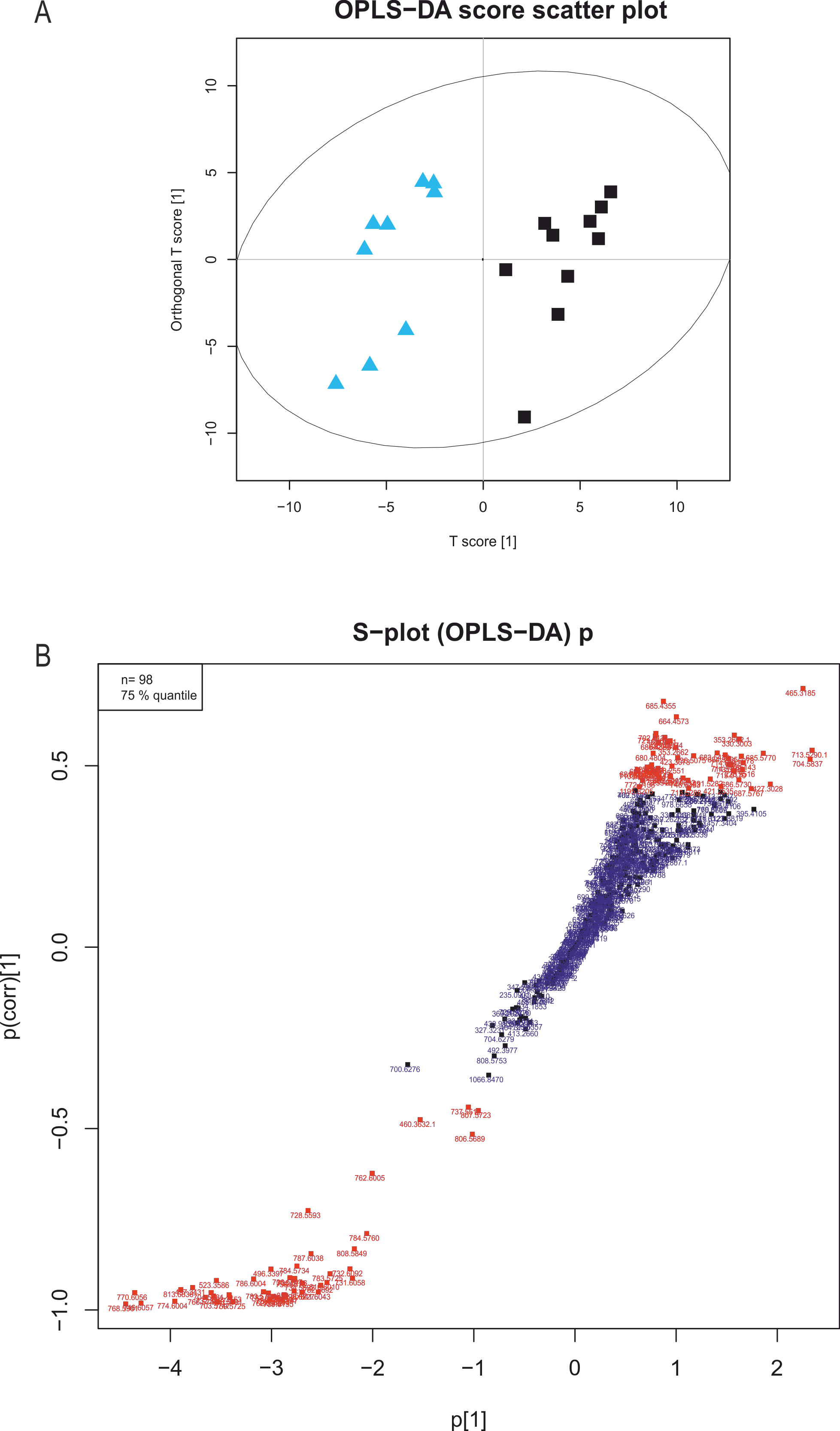


Figure S5: (A) OPLS-DA score scatter plot and (B) S-plot build for untargeted metabolomic analysis of CSF samples (transgenic rats - blue triangles; controls - black squares).


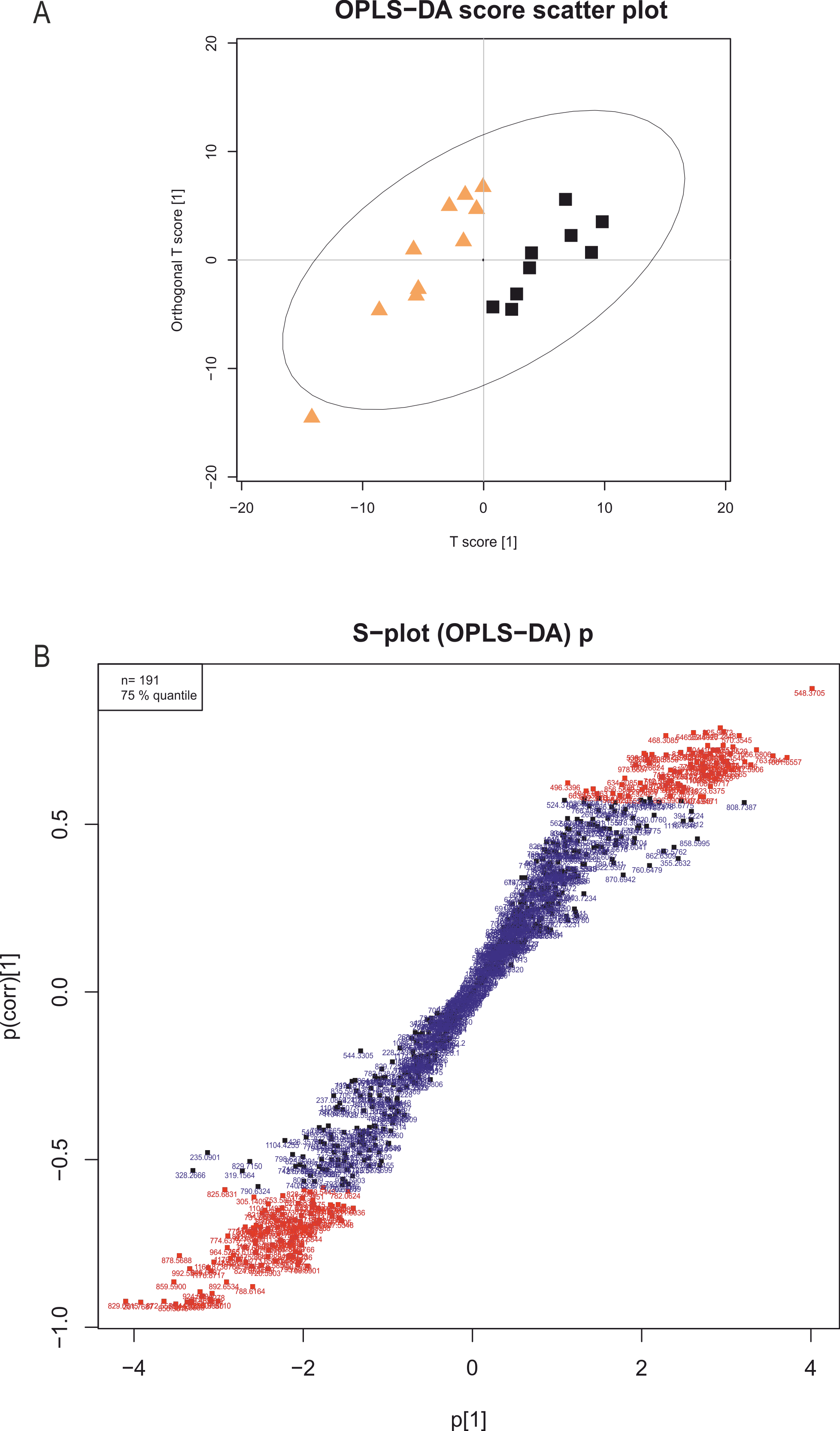


Figure S6: (A) OPLS-DA score scatter plot and (B) S-plot build for untargeted metabolomic analysis of plasma samples (transgenic rats - orange triangles; controls - black squares).


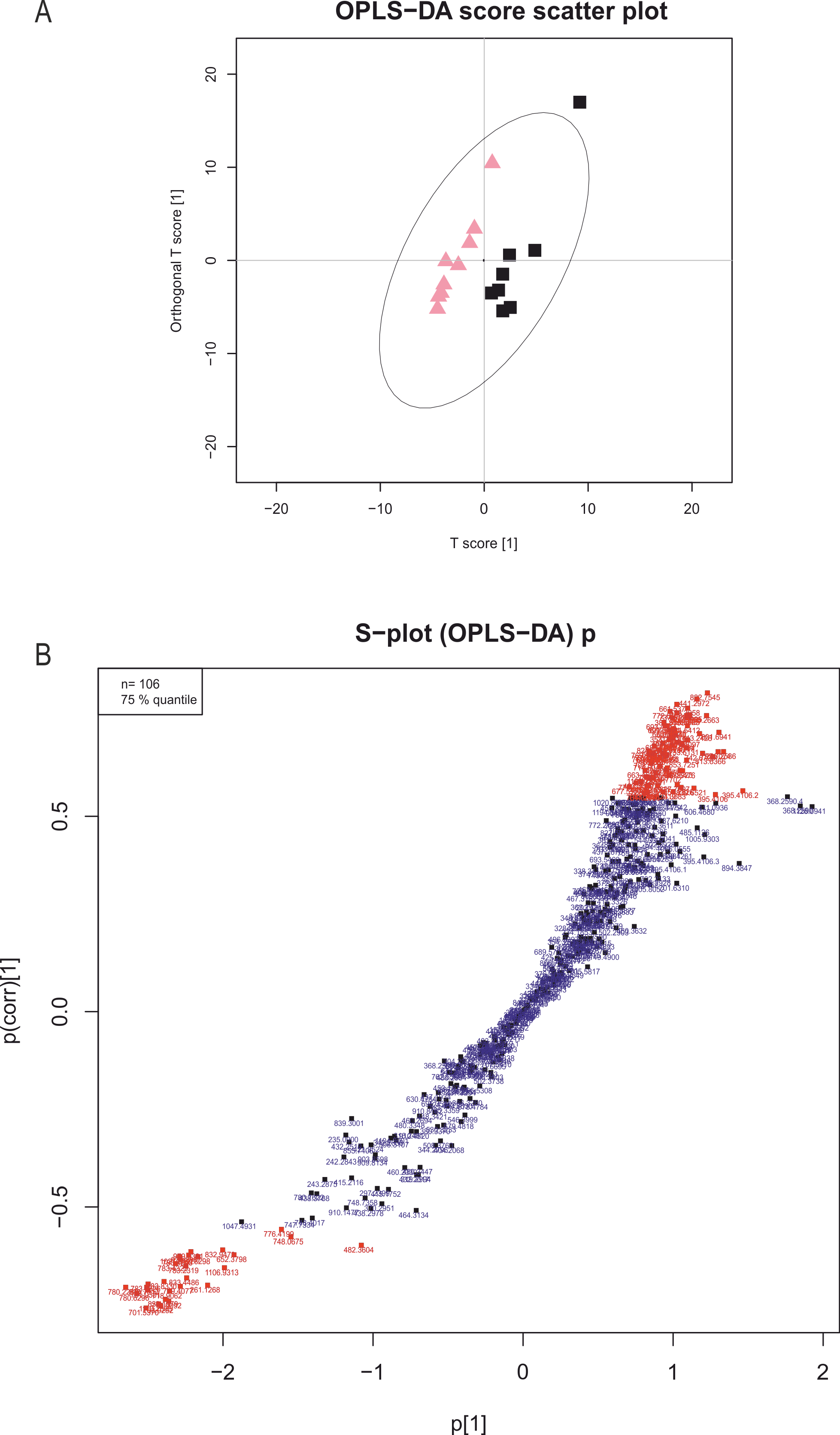


Figure S7: (A) OPLS-DA score scatter plot and (B) S-plot build for untargeted metabolomic analysis of brain samples (transgenic rats -pink triangles; controls - black squares).


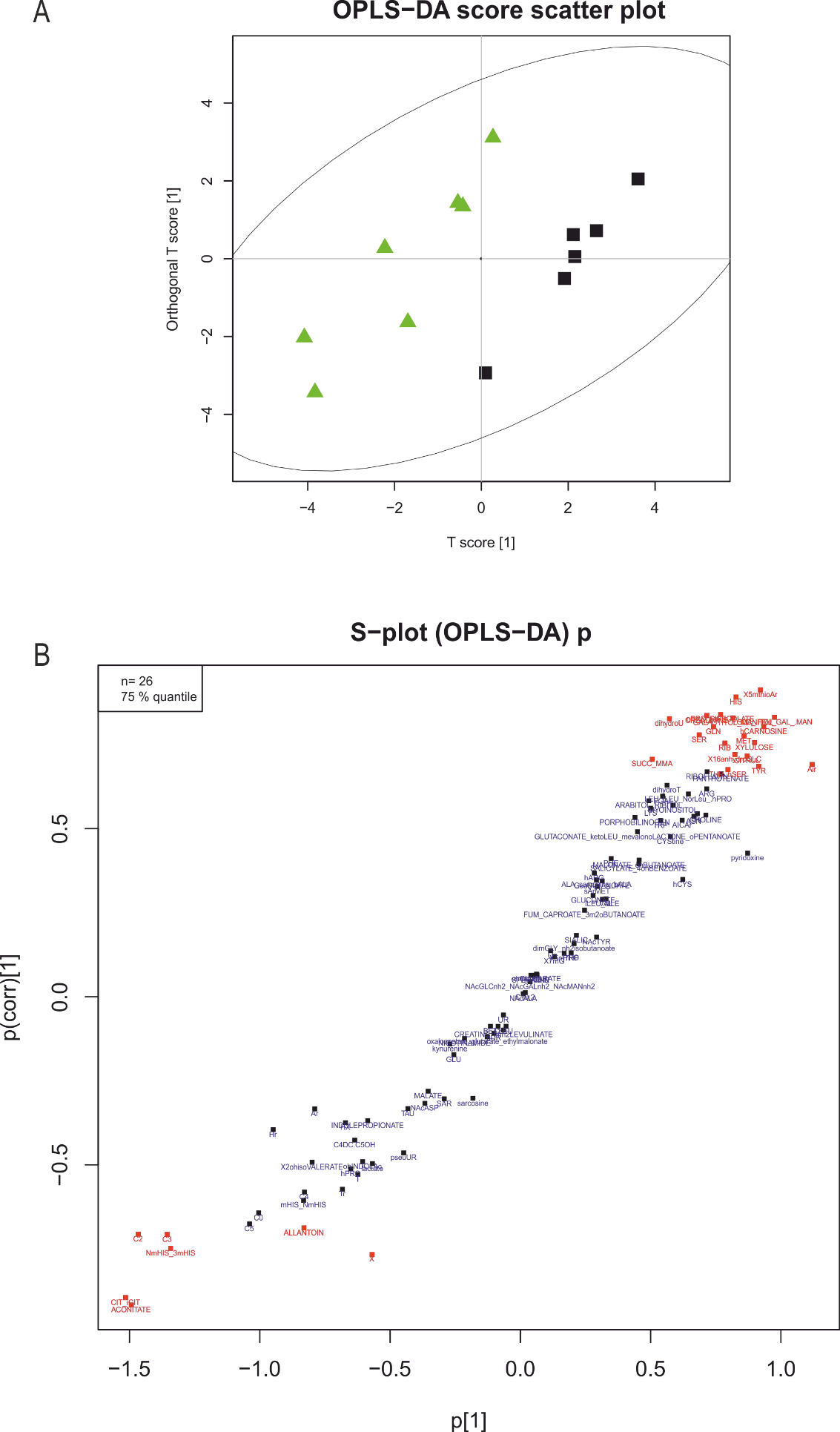


Figure S8: Confirmation study. (A) OPLS-DA score scatter plot and (B) S-plot build for targeted metabolomic analysis of CSF (transgenic rats - green triangles; controls - black squares).


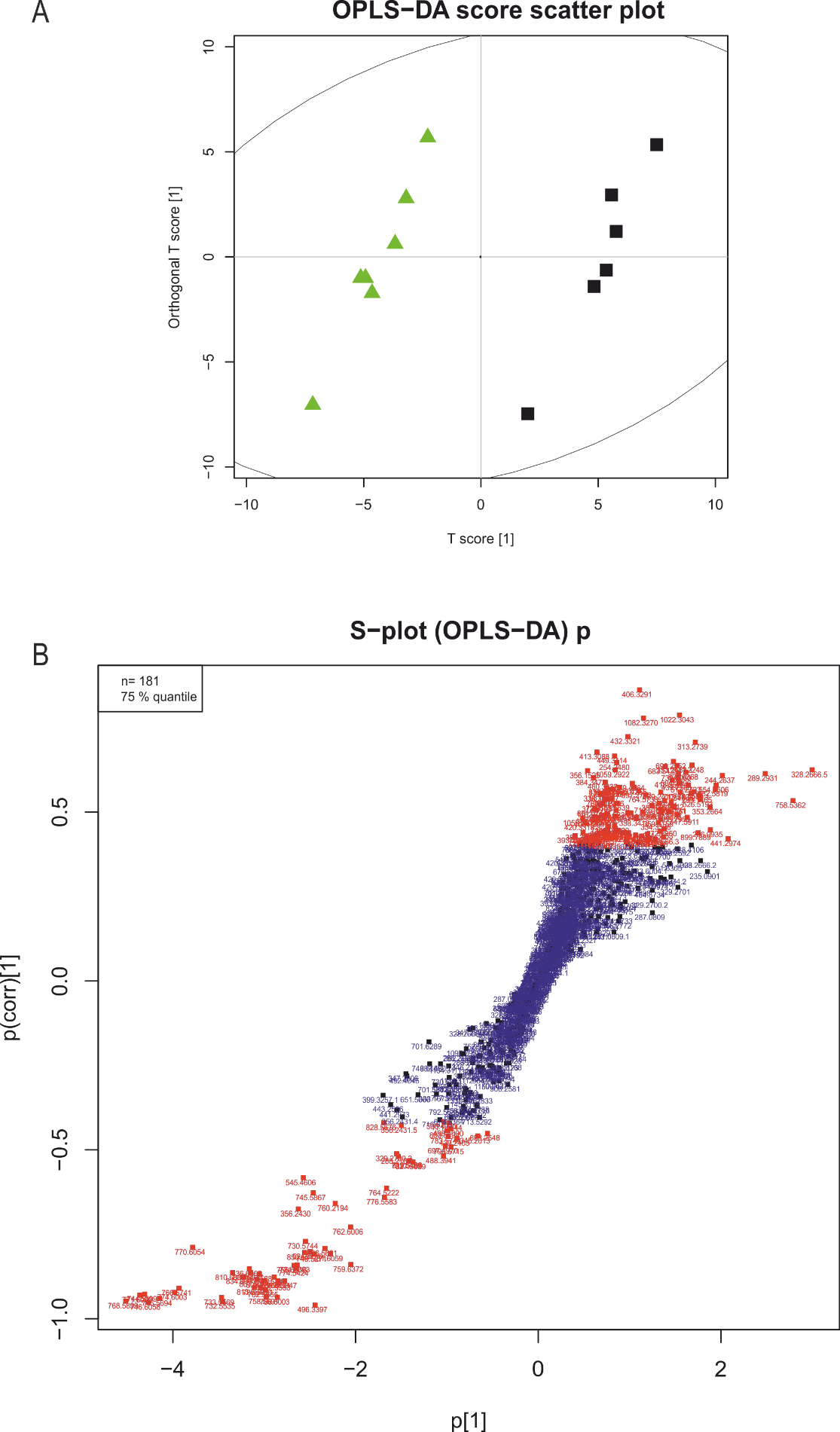


Figure S9: Confirmation study. (A) OPLS-DA score scatter plot and (B) S-plot build for untargeted metabolomic analysis of CSF (transgenic rats - green triangles; controls - black squares).

Table S4: Untargeted analysis of brain tissue samples. Twenty the most discriminating features from OPLS-DA analysis (sorted by absolute value of pcorr1 axis – variation related to variable magnitude). P-value, fold change and corrected α value (after Bonferroni correction) for these features are shown.

| *m/z* | p1 | pcorr1 | fold change | t-test  p value  (α =1.18E-04) ^‡^ |
| --- | --- | --- | --- | --- |
|  |  |  |  |  |
| 822.7545 | 1.23 | 0.82 | 1.12 | 8.82E-02 |
| 441.2972 | 1.16 | 0.80 | 1.08 | 1.10E-01 |
| 661.5377 | 1.02 | 0.79 | 1.08 | 2.94E-02 |
| 533.3058 | 1.10 | 0.78 | 1.02 | 9.16E-02 |
| 772.6140 | 0.98 | 0.77 | 1.13 | 5.46E-02 |
| 737.5718 | 1.02 | 0.76 | 1.04 | 1.91E-01 |
| 732.5985 | 1.11 | 0.76 | 1.07 | 2.48E-01 |
| 701.5370 | -2.51 | -0.76 | 0.76 | 2.17E-01 |
| 381.2974 | 1.09 | 0.76 | 1.08 | 1.18E-01 |
| 353.2663 | 1.22 | 0.76 | 1.09 | 6.44E-02 |
| 298.3467 | 1.11 | 0.75 | 1.19 | 2.49E-02 |
| 656.5825 | 1.09 | 0.75 | 1.13 | 4.78E-02 |
| 783.9292 | -2.42 | -0.75 | 0.65 | 1.16E-01 |
| 383.3155 | 1.00 | 0.75 | 1.06 | 1.51E-01 |
| 1110.2595 | -2.43 | -0.75 | 1.02 | 2.49E-01 |
| 719.9092 | -2.36 | -0.74 | 0.68 | 5.16E-02 |
| 693.4544 | 0.94 | 0.74 | 1.06 | 2.12E-01 |
| 833.1979 | -2.38 | -0.74 | 0.82 | 2.44E-01 |
| 677.5514 | 0.96 | 0.73 | 1.07 | 2.25E-01 |
| 741.5412 | 1.10 | 0.73 | 1.08 | 1.40E-01 |


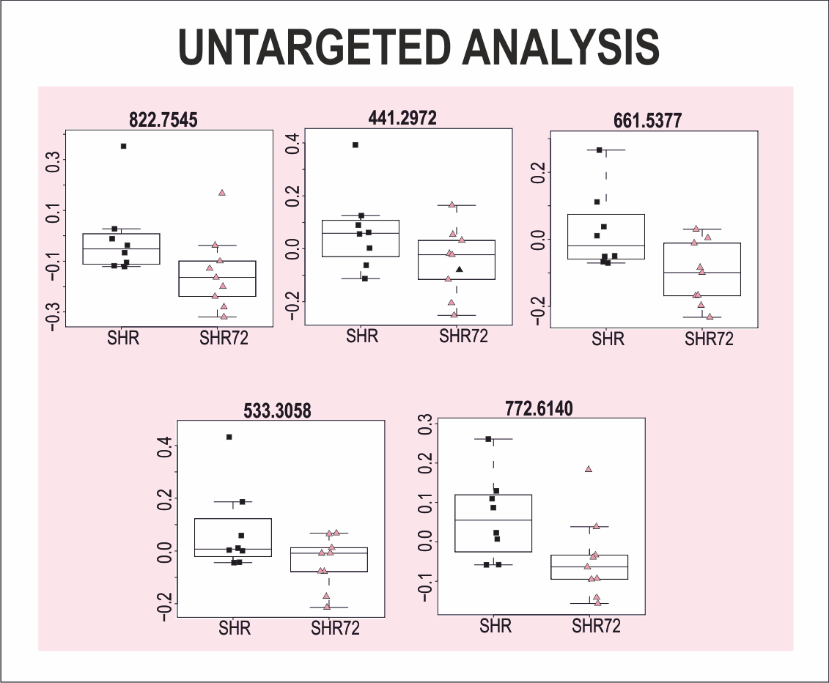


Figure S10: Boxplots of most discriminating features from brain tissue samples of transgenic rats (pink triangles) and controls (black squares) from untargeted metabolomic analysis.
